# Comparative proteomic analysis provides new insights into regulation of microspore embryogenesis induction in winter triticale (× *Triticosecale* Wittm.) after 5-azacytidine treatment

**DOI:** 10.1038/s41598-021-01671-y

**Published:** 2021-11-15

**Authors:** Monika Krzewska, Ewa Dubas, Gabriela Gołębiowska, Anna Nowicka, Agnieszka Janas, Kamil Zieliński, Ewa Surówka, Przemysław Kopeć, Przemysław Mielczarek, Iwona Żur

**Affiliations:** 1 The Franciszek Górski Institute of Plant Physiology Polish Academy of Sciences, Niezapominajek 21, 30-239 Kraków, Poland; 2 Chair of Genetics, Institute of Biology, Pedagogical University of Krakow, Podchorążych 2, 31-084 Kraków, Poland; 3 Institute of Experimental Botany of the Czech Academy of Sciences, Centre of the Region Haná for Biotechnological and Agricultural Research, Šlechtitelů 31, 779 00 Olomouc, Czech Republic; 4 Department of Analytical Chemistry and Biochemistry, Faculty of Materials Science and Ceramics, AGH University of Science and Technology, Mickiewicza 30 ave., 30-059 Kraków, Poland

**Keywords:** Plant sciences, Plant stress responses

## Abstract

Effective microspore embryogenesis (ME) requires substantial modifications in gene expression pattern, followed by changes in the cell proteome and its metabolism. Recent studies have awakened also interest in the role of epigenetic factors in microspore de-differentiation and reprogramming. Therefore, demethylating agent (2.5–10 μM 5-azacytidine, AC) together with low temperature (3 weeks at 4 °C) were used as ME-inducing tiller treatment in two doubled haploid (DH) lines of triticale and its effect was analyzed in respect of anther protein profiles, expression of selected genes (*TAPETUM DETERMINANT1 (TaTPD1-like), SOMATIC EMBRYOGENESIS RECEPTOR KINASE 2 (SERK2)* and *GLUTATHIONE S-TRANSFERASE* (*GSTF2*)) and ME efficiency. Tiller treatment with 5.0 µM AC was the most effective in ME induction; it was associated with (1) suppression of intensive anabolic processes-mainly photosynthesis and light-dependent reactions, (2) transition to effective catabolism and mobilization of carbohydrate reserve to meet the high energy demand of cells during microspore reprograming and (3) effective defense against stress-inducing treatment, i.e. protection of proper folding during protein biosynthesis and effective degradation of dysfunctional or damaged proteins. Additionally, 5.0 µM AC enhanced the expression of all genes previously identified as being associated with embryogenic potential of microspores (*TaTPD1-like, SERK* and *GSTF2*).

## Introduction

Microspores are male gametophytic plant cells evolutionarily programmed to develop into pollen grains. However, under specific circumstances, microspores initiate an alternative developmental pathway and generate zygotic-like embryos (embryo-like structures, ELS) capable of regenerating whole plants^1^. This fascinating process is based on the phenomenon of ‘cell totipotency’ and is called ‘microspore embryogenesis’ (ME). Abiotic stress factors such as extreme temperature, starvation or osmotic shock can be used as a trigger of ME, either alone or in various combinations^2,3^. ME is a perfect in vitro system to study regulatory mechanisms of cell reprogramming, dedifferentiation and early embryogenesis, while omitting difficulties associated with dissection of zygotes and immature embryos, which, *in planta*, are surrounded by maternal tissues^4^. Moreover, ME is a powerful biotechnological tool applied in crop breeding for the rapid production of completely homozygous doubled haploid (DH) plants. DHs are also very useful as models for basic research, mapping population production, genetic selection and screening for recessive alleles.

Each year, some modifications of DH production procedures in different plants species are reported^5^; however, the effectiveness of the process in many cases, including triticale, is still not satisfactory. Low efficiency of microspore reprogramming, abortion of multicellular structures after sporoderm wall rupture and high frequency of albino plant regeneration belong to the most often reported bottlenecks^4,6^. Slow rate of the progress is associated with a very complex interaction networks between endogenous and environmental factors that determine the successful initiation of ME. Among them, physiological condition of donor plants, ME-inducing treatment parameters and, obviously, in vitro culture conditions seem to be the most important. However, some factors more difficult to interpret like seasonal effect, should also be taken into consideration^7^.

Reprogramming of the microspore and initiation of embryogenesis involve genome-wide changes of gene expression that allow the cell fate program to be halted and switched to a new developmental pathway^8^. Recently harnessed advanced methods of wide transcriptome analysis allowed the selection of differentially expressed genes during ME initiation and ELS development^9–12^. Majority of them has been identified as being involved in cell protection against stress, sugar metabolism, signaling and proteolysis. The first and comprehensive RNA-seq analysis on wheat microspores also revealed a group of up-regulated genes controlling chromatin modifications and cell component organization^11^.

The analysis of triticale DH lines with different ME efficiency resulted in the identification of several genes associated with microspore embryogenic potential^12^. The same genes were previously associated with efficient wheat microspores reprogramming and ME initiation^13^.

Although transcriptomic data are very valuable, they do not reveal the full complexity of the ME mechanism. Many molecular and bioinformatics studies have shown that mRNA and protein abundances are not always correlated^14^. One of the reasons for this discrepancy is the occurrence of various processes after mRNA synthesis, such as post-transcriptional, translational and protein degradation regulation. Therefore, the data obtained from proteomic analyses are a highly anticipated supplement, expanding our understanding of many physiological processes, including ME.

Only a few studies examined this aspect of microspore reprogramming and proposed some candidate proteins possibly involved in ME induction^15–17^.

The first two potential ME protein markers, identified in oilseed rape, were storage proteins, 12S glycoprotein^18^ and napine^19^. Also in oilseed rape, Cordewener et al.^15^ indicated proteins involved in antioxidant defense as associated with ME, others-heat shock proteins (HSP70 and HSP90)-were identified also in *Nicotiana tabacum* and *Capsicum annuum*^20,21^. In maize, Uváčková et al.^17^ identified 19 unique proteins, mainly involved in metabolism, protein synthesis and cell wall remodeling as correlated with ME induction. A similar analysis on winter triticale^16^ revealed 31 proteins involved in the determination of microspore competence for embryogenesis, stress response and in the regulation of ME induction. These results suggested that the competence of microspore to embryogenic development requires a sufficient energy supply and an efficient cell protection to survive under long-term low temperature treatment (3 weeks at 4 °C), conventionally used to induce ME in triticale. The present study is a continuation of our earlier research, including the latest reports on the role of epigenetic factors in ME induction and regulation.

It is assumed that the totipotency of cells is associated with an open chromatin conformation, which is manifested by large nuclei and homogenous euchromatin^22^. Changes in global genome organization and epigenetic modifications are the key factors in genome flexibility that are also involved in stress-induced plant cell reprogramming^23^. The role of DNA methylation in development has been studied using a nucleotide analogue with DNA demethylation activity, 5-azacytidine (AC). AC is randomly incorporated into the newly synthesized DNA strand instead of cytosine, resulting in a decreased activity of DNA methyltransferase, followed by genome hypomethylation^24^. Changes in global DNA methylation level associated with ME induction have been observed in various plant species^25,26^. The above reports have supported the hypothesis that DNA hypomethylation is essential for the regulation of chromatin conformation and the shift of the gene expression program. Therefore, AC treatment has been used to promote the processes of embryogenesis in somatic^27^ and gametophytic cells^23^. As an example, in our previous study^26^, treatments with two cytosine-analogs: AC and 2′-deoxy-5-azacytidine (DAC) induced changes in DNA methylation levels, changed expression of some ME-associated genes (*TaTPD1-like, GSTF2, GSTA2, CHI3, Tad1, TaNF-YA7, SERK2, TaME1*) and influenced on ME effectiveness. Both drugs showed significant cytotoxicity in a dose-dependent manner with the most promising results obtained after application of 10°µM AC during low temperature treatment of tillers. To understand better the role of DNA methylation in the acquisition of microspores responsiveness to embryogenesis induction, the same plant material (two DH lines of triticale significantly different with respect to embryogenic potential) and similar experimental scheme were used in this study with the main aim to identify changes in protein profile associated with DNA methylation inhibitor treatment and to provide new insights into ME induction in winter triticale. Additionally, we determined the transcript levels of genes associated with DNA methylation (*TaMET2B*), intra-embryonic communication (*TaTPD1-like*), signaling (*SERK2*) and defense reactions (*GSTF2*). The genes were selected on the basis of previous experiments^26^ as showing the most divergent expression patterns after treatment with DNA methylation inhibitors.

## Results

### Microspore embryogenesis effectiveness in anther cultures after 5-azacytidine treatment

**Figure 1:**
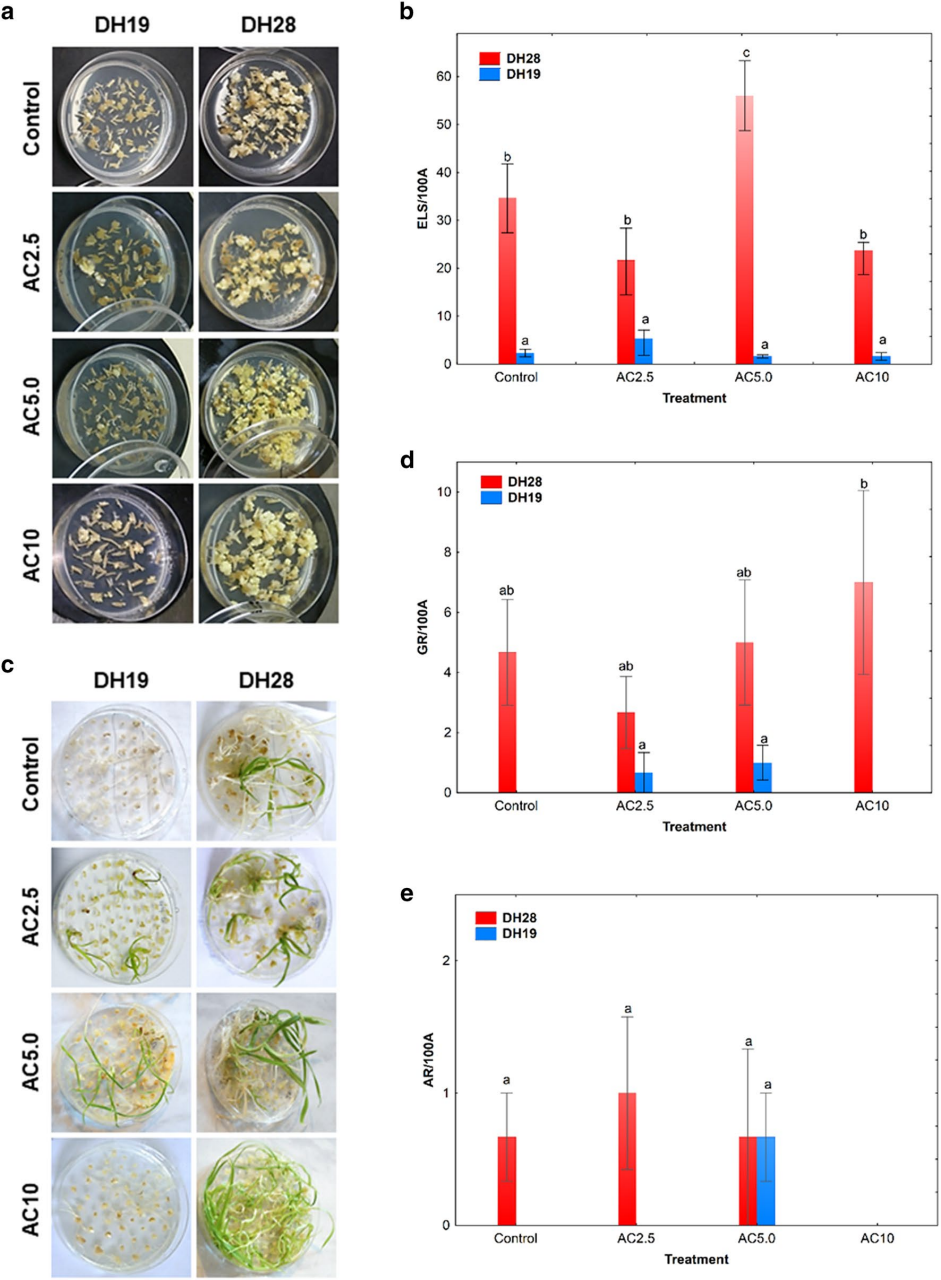
The effect of 5-azacytidine (AC) on the effectiveness of embryo-like structure production (ELS; **a**, **b**) and plant regeneration (**c**–**e**) in anther cultures of two doubled haploid (DH) lines of triticale, significantly differed in their responsiveness to microspore embryogenesis (responsive DH28 and recalcitrant DH19). AC was applied at concentrations of 2.5 µM (AC2.5), 5.0 µM (AC5.0) and 10 µM (AC10) during low temperature tillers treatment. (**a**) ELS formation after 6 weeks of in vitro culture; (**b**) mean number of ELS per 100 anthers (ELS/100A); (**c**) plant regeneration after 10 weeks of in vitro culture; (**d**) mean number of green plants per 100 anthers (GR/100A); (**e**) mean number of albino plants per 100 anthers (AR/100A). Means calculated from at least three biological replicates (petri dish containing 100 anthers collected from one spike). Error bars denote standard error of the mean (SE). Data marked with the same letter do not differ according to Duncan’s multiple range test (*p* ≤ 0.05).

Two studied DH lines significantly differed in their responsiveness to ME-inducing treatment (Fig. 1a–e) which was significantly influenced (*p* ≤ 0.001) by all tested variables: the genotype of a donor plant, the type of tiller treatment and their interaction (Supplementary Table S1). In result of standard low temperature tiller treatment (3 weeks at 4 °C), the responsive line DH28 was characterized by 15-times higher mean value of the number of ELS per 100 anthers (ELS/100A) in comparison to the recalcitrant line DH19 (Fig. 1b). The most effective induction of ME for DH28 was obtained when standard tiller treatment was combined with 5.0 µM AC application (AC5.0). After this treatment, the number of ELS increased significantly (1.7-fold) in comparison to the control (Fig. 1b). Unfortunately, recalcitrance of DH19 was not overcome efficiently by any of the applied treatment and the number of produced ELS did not exceed 6 ELS/100A (Fig. 1a,b).

Plant regeneration after standard low temperature treatment of tillers was observed only in anther cultures of responsive line DH28 (Fig. 1c–e). Although chemically-induced DNA demethylation had no significant effect on plant regeneration in both DH lines, the highest number of green plants per 100 anthers (7 GR/100A) was obtained in responsive DH28 line following the treatment with the highest AC concentration (10 µM; AC10) (Fig. 1c,d). This treatment also completely blocked the albino plants formation in DH28 (Fig. 1c,e). Even though the treatments with AC5.0 and AC2.5 (2.5 µM 5-azacytidine) resulted in green plant regeneration of the recalcitrant line DH19, the effectiveness of this process was very low (up to 1 GR/100A). Moreover, AC5.0 treatment caused the regeneration of some albino plants in DH19 (Fig. 1d,e).

Summarizing, the application of 5.0 µM AC during standard ME-inducing tillers treatment highly increased the efficiency of ELS formation in anther cultures of responsive line DH28. A positive tendency was observed in respect of green plant regeneration capacity after 10 µM AC application in DH28, whereas the AC2.5 and AC5.0 treatments caused some plant regeneration in anther cultures of DH19.

### Effect of 5-azacytidine on anther protein profiles

Total protein yields obtained using the phenol-based protocol ranged from 0.5 to 2.2 mg per 1 g FW and were distinctly lower in AC10-treated anthers of both studied triticale DH lines (Supplementary Table S2).

In total, of 1540 quantitatively different protein spots detected after 2D gel electrophoresis, 850 showed at least a twofold change in their abundance after AC treatments and were classified as differentially abundant proteins (DAPs) (Supplementary Fig. S1).

**Figure 2:**
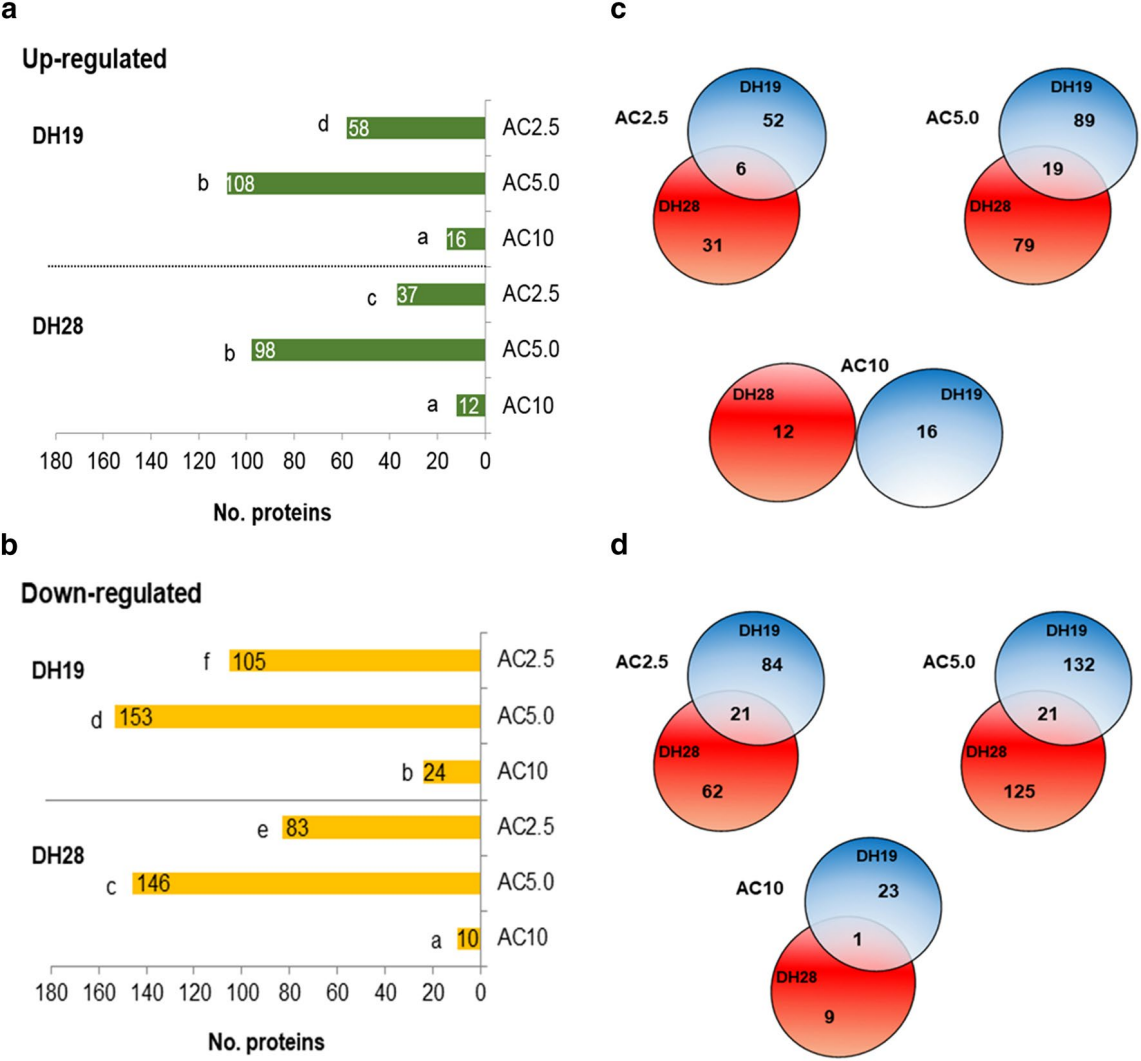
Changes induced by 5-azacytitidine (AC) tiller treatment in anther protein profiles (at least ± 2-fold change, *p* ≤ 0.05) of two doubled haploid (DH) lines of triticale, significantly differed in their responsiveness to microspore embryogenesis (responsive DH28 and recalcitrant DH19). AC was applied at concentrations of 2.5 µM (AC2.5), 5.0 µM (AC5.0) and 10 µM (AC10) during low temperature tillers treatment. Number of up-regulated (**a**) and down-regulated (**b**) proteins after AC treatment in comparison to the control (3 weeks at 4 °C); (**c**, **d**) Venn diagrams shows the overlap of the differentially expressed proteins following AC treatment. Quantitative Venn diagrams were constructed using a web application BioVenn accessible at http://www.biovenn.nl/index.php, but the original colours of the graphs were modified.

Generally, many more proteins were down-regulated than up-regulated after AC treatment in both studied DH lines (Fig. 2a,b). The genotype of the donor plant and the type of tiller treatment had a significant influence on the number of DAPs (Supplementary Table S3). Only 0–21 protein spots (up to 12%) changed similarly in both DH lines (Fig. 2c,d). Interestingly, the highest number of DAPs (261 in DH19 and 244 in DH28) was observed after AC5.0 treatment, whereas the highest AC concentration (10 μM) caused the smallest changes in anther protein profiles (40 and 22 in DH19 and DH28, respectively) (Fig. 2a,b).

**Figure 3:**
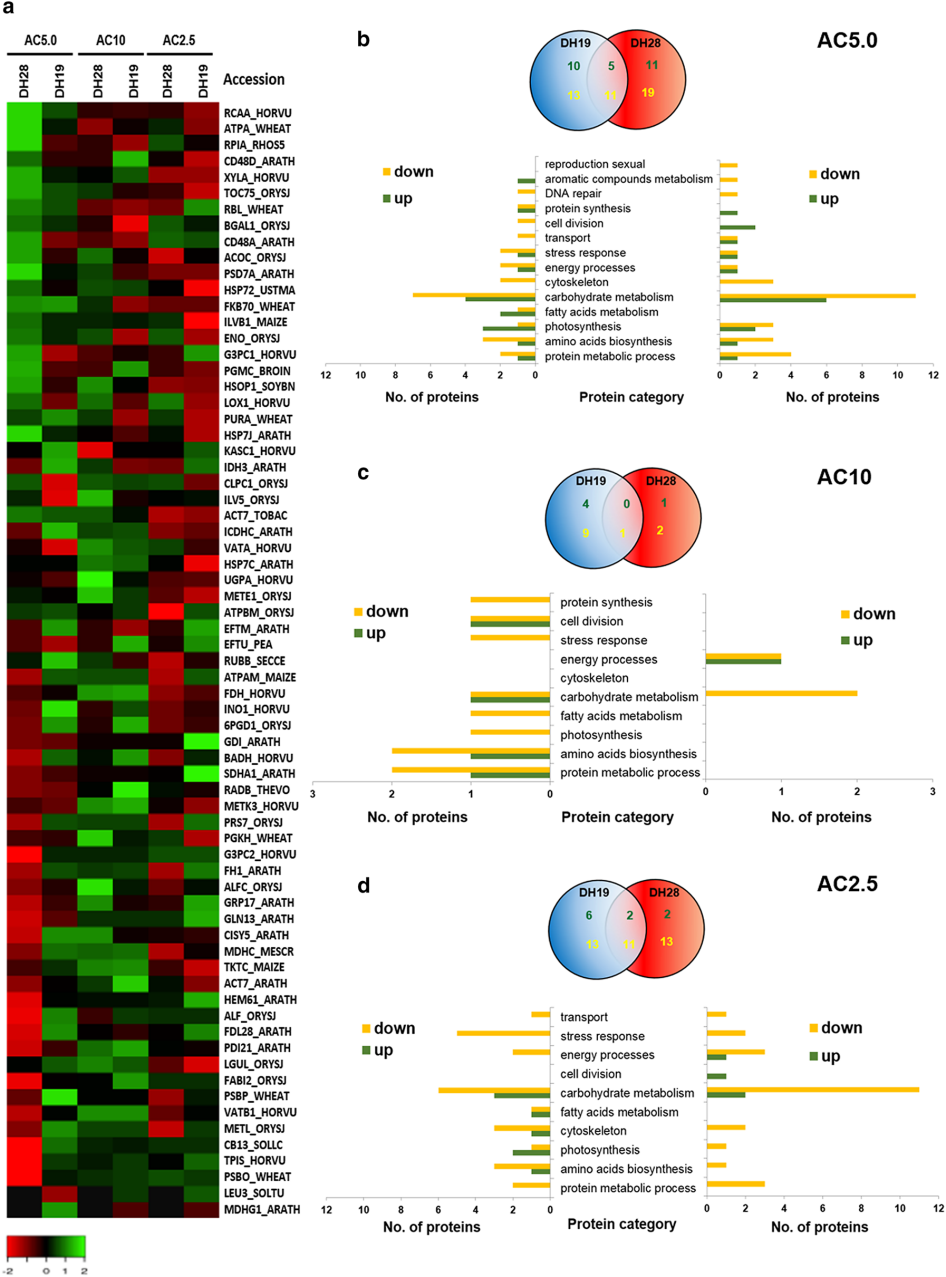
Effect of 5-azacytidine (AC) on protein abundance identified in the anthers of two doubled haploid (DH) lines of triticale, significantly differed in their responsiveness to microspore embryogenesis (responsive DH28 and recalcitrant DH19). AC was applied at concentrations of 2.5 µM (AC2.5), 5.0 µM (AC5.0) and 10 µM (AC10) during low temperature tillers treatment. (**a**) Heat map showing differentially expressed proteins identified after AC treatment. The heatmap was constructed using Heatmapper accessible at http://www.heatmapper.ca/expression/. Colours correspond to the log transformed values of protein fold-change. Accession numbers of identified proteins are presented in Supplementary Table S4. The proteins were ordered according to the fold change after AC5.0 treatment in the responsive line DH28. (**b**–**d**) Venn diagrams shows the number and overlap of protein spots showing quantitative changes in their abundance in comparison to the control (3 weeks at 4 °C) after AC5.0 (**b**), AC10 (**c**) and AC2.5 (**d**) treatments while graphs depicts their functional classification. Quantitative Venn diagrams were constructed using a web application BioVenn accessible at http://www.biovenn.nl/index.php, but the original colours of the graphs were modified. Green font colour is used for the number of up-regulated and yellow font for down-regulated proteins.

From all DAPs, 69 were successfully identified using the nano LC–MS/MS method (Supplementary Table S4, Fig. 3a, Supplementary Fig. S1). The majority of them (almost 60%) was involved in various metabolic processes, such as carbohydrate and protein metabolism, photosynthesis or amino acid biosynthesis (Supplementary Fig. S2). Lower concentrations of DNA methylation inhibitor (2.5 μM and 5.0 μM) significantly changed the accumulation of proteins associated with carbohydrate metabolism (Fig. 3b,d), whereas the AC10 treatment mainly decreased the abundance of proteins involved in protein metabolic processes in the recalcitrant line DH19 (Fig. 3c).

Almost all observed changes were quantitative in nature, except for two protein spots (SSP1101, SSP2105) in the anther proteome of the responsive line DH28. The first-protein species no. 1101, identified as malate dehydrogenase, glyoxysomal was repressed, whereas the second, protein no. 2105 (3-isopropylmalate dehydrogenase, chloroplastic) was induced after all AC treatments (Fig. 3a, Table 1).

**Table 1:** A list of anther proteins, which accumulation significantly changed after microspore embryogenesis (ME) inducing triticale tillers treatment combining low temperature (3 weeks at 4 °C) with the application of 5.0 µM of 5-azacytidine, DNA demethylating agent.

No.	Spot no.	Protein name	Accession	MW^a^ (kDa)	pI^b^	Protein score^c^	Seq. Cov [%]^d^	Reference organism	Protein function	Fold change^e^	
										DH28	DH19
**Qualitative changes**											
1	2105	3-Isopropylmalate dehydrogenase, chloroplastic	LEU3_SOLTU	39.7	9.2	147.5	7.3	*Solanum tuberosum*	Amino acids biosynthesis	New	− 1.2
2	1101	Malate dehydrogenase, glyoxysomal	MDHG1_ARATH	37.3	9.2	42.7	3.1	*Arabidopsis thaliana*	Fatty acids metabolism	Dissap	2.0
**Quantitative changes**											
Up-regulated in responsive line (DH28) and down-regulated/not significantly changed in recalcitrant line (DH19)*											
3	7707	Acetolactate synthase 1, chloroplastic	ILVB1_MAIZE	68.9	6.8	57.1	3.4	*Zea mays*	Amino acids biosynthesis	2.1	− 1.7
4	6716	Ribose-5-phosphate isomerase A	RPIA_RHOS5	27.0	4.7	45.2	3.8	*Rhodobacter sphaeroides*	Carbohydrate metabolism	4.9	− 3.1
5	7512	Xylose isomerase	XYLA_HORVU	53.6	5.2	460.2	19.0	*Hordeum vulgare*	Carbohydrate metabolism	4.7	1.7
6	3102	Glyceraldehyde-3-phosphate dehydrogenase 2, cytosolic	G3PC2_HORVU	36.5	6.8	111.0	9.2	*Hordeum vulgare*	Carbohydrate metabolism	3.0	1.3
7	5904	Putative aconitate hydratase, cytoplasmic	ACOC_ORYSJ	98.0	5.6	89.6	1.1	*Oryza sativa* subsp*. japonica*	Carbohydrate metabolism	2.9	− 2.0
8	7402	Enolase	ENO_ORYSJ	47.9	5.3	361.0	17.7	*Oryza sativa* subsp*. japonica*	Carbohydrate metabolism	2.1	− 1.1
9	6902	Cell division control protein 48 homolog D	CD48D_ARATH	90.3	4.9	186.7	5.2	*Arabidopsis thaliana*	Cell division	4.8	1.4
10	8903	Cell division control protein 48 homolog A	CD48A_ARATH	89.3	5.0	122.3	5.7	*Arabidopsis thaliana*	Cell division	3.0	− 2.3
11	2203	26S proteasome non-ATPase regulatory subunit 7 homolog A	PSD7A_ARATH	34.7	6.0	201.1	12.7	*Arabidopsis thaliana*	Protein metabolic process	2.3	1.5
12	9613	Heat shock 70 kDa protein 2	HSP72_USTMA	70.3	4.9	282.7	9.5	*Ustilago maydis*	Stress response	2.3	− 2.1
13	601	Protein TOC75, chloroplastic	TOC75_ORYSJ	87.6	9.2	211.8	6.7	*Oryza sativa* subsp. *japonica*	Transport	4.5	1.7
Down-regulated in responsive line (DH28) and up-reguated/not significantly changed in recalcitrant line (DH19)*											
14	4507	Betaine aldehyde dehydrogenase	BADH_HORVU	54.3	5.8	129.9	5.9	*Hordeum vulgare*	Amino acids biosynthesis	− 2.3	2.9
15	7111	Glutamine synthetase cytosolic isozyme 1–3	GLN13_ARATH	38.6	5.7	100.9	10.2	*Arabidopsis thaliana*	Amino acids biosynthesis	− 3.3	− 1.3
16	6102	Coproporphyrinogen-III oxidase 1, chloroplastic	HEM61_ARATH	43.8	6.2	176.9	5.7	*Arabidopsis thaliana*	Aromatic compounds metabolism	− 4.9	− 1.4
17	6403	Inositol-3-phosphate synthase	INO1_HORVU	56.1	5.4	449.5	15.9	*Hordeum vulgare*	Carbohydrate metabolism	− 2.2	3.7
18	1103	Fructose-bisphosphate aldolase cytoplasmic isozyme	ALF_ORYSJ	36.7	9.0	239.3	8.9	*Oryza sativa* subsp*. japonica*	Carbohydrate metabolism	− 5.0	2.6
19	5103	Malate dehydrogenase, cytoplasmic	MDHC_MESCR	35.5	6.0	389.3	12.0	*Oryza sativa* subsp*. japonica*	Carbohydrate metabolism	− 3.7	1.3
20	1304	Citrate synthase 5, mitochondrial	CISY5_ARATH	51.7	6.2	203.8	6.7	*Arabidopsis thaliana*	Carbohydrate metabolism	− 3.6	1.1
21	4606	Succinate dehydrogenase [ubiquinone] flavoprotein subunit 1, mitochondrial	SDHA1_ARATH	69.6	5.8	223.4	7.6	*Arabidopsis thaliana*	Carbohydrate metabolism	− 2.3	− 1.6
22	5305	6-Phosphogluconate dehydrogenase, decarboxylating 1	6PGD1_ORYSJ	52.7	5.8	229.7	6.5	*Oryza sativa* subsp*. japonica*	Carbohydrate metabolism	− 2.2	1.2
23	7002	Triosephosphate isomerase, cytosolic	TPIS_HORVU	26.7	5.3	603.2	30.4	*Hordeum vulgare*	Carbohydrate metabolism	− 19.3	1.1
24	5106	Formin-like protein 1	FH1_ARATH	115.1	9.3	50.4	1.5	*Arabidopsis thaliana*	Cytoskeleton	− 3.0	1.6
25	5508	ATP synthase subunit alpha, mitochondrial	ATPAM_MAIZE	55.1	5.8	232.2	15.2	*Zea mays*	Energy processes	− 2.0	1.4
26	5007	Oxygen-evolving enhancer protein 2, chloroplastic	PSBP_WHEAT	27.3	9.5	855.9	44.6	*Triticum aestivum*	Photosynthesis	− 6.7	17.7
27	8002	Chlorophyll a-b binding protein 8, chloroplastic	CB13_SOLLC	24.2	0.0	130.3	5.5	*Solanum lycopersicum*	Photosynthesis	− 12.8	1.6
28	1002	Putative F-box/FBD/LRR-repeat protein At4g26350	FDL28_ARATH	49.6	9.7	40.4	2.1	*Arabidopsis thaliana*	Protein metabolic process	− 5.6	2.1
29	3305	26S protease regulatory subunit 7	PRS7_ORYSJ	47.7	5.9	194.2	11.0	*Oryza sativa* subsp.* japonica*	Protein metabolic process	− 2.7	1.3
30	0002	Probable inactive methyltransferase	METL_ORYSJ	40.0	5.6	60.2	5.1	*Oryza sativa* subsp.* japonica*	Protein metabolic process	− 7.3	1.2
31	3502	Oleosin GRP-17	GRP17_ARATH	53.2	10.9	90.0	5.3	*Arabidopsis thaliana*	Reproduction sexual	− 3.2	− 1.5
32	7004	Lactoylglutathione lyase	LGUL_ORYSJ	32.5	5.4	326.8	11.3	*Oryza sativa* subsp.* Japonica*	Stress response	− 6.2	− 1.3
Up-regulated in both DH lines with higher fold change in DH28***											
33	3904	Beta-galactosidase 1	BGAL1_ORYSJ	91.7	5.7	140.5	4.4	*Oryza sativa* subsp.* japonica*	Carbohydrate metabolism	3.0	2.3
34	2503	ATP synthase subunit alpha, chloroplastic	ATPA_WHEAT	55.3	6.1	279.3	11.7	*Triticum aestivum*	Energy processes	5.7	2.0
35	8202	Ribulose bisphosphate carboxylase/oxygenase activase A, chloroplastic	RCAA_HORVU	51.0	8.9	627.4	17.2	*Hordeum vulgare*	Photosynthesis	6.3	2.6
36	2409	Ribulose bisphosphate carboxylase large chain	RBL_WHEAT	52.8	6.2	910.3	19.3	*Triticum aestivum*	Photosynthesis	3.4	2.3
Down-regulated in both DH lines with higher fold change in DH28****											
37	8103	Fructose-bisphosphate aldolase, chloroplastic	ALFC_ORYSJ	42.0	6.5	490.9	22.2	*Oryza sativa* subsp. japonica	Carbohydrate metabolism	− 3.1	− 2.0
38	7706	Transketolase, chloroplastic	TKTC_MAIZE	72.9	5.4	72.6	1.9	*Zea mays*	Carbohydrate metabolism	− 3.8	− 2.4
39	8307	Actin-7	ACT7_ARATH	41.7	5.2	442.4	30.2	*Arabidopsis thaliana*	Cytoskeleton	− 4.1	− 2.0
40	7001	Enoyl-[acyl-carrier-protein] reductase [NADH] 2, chloroplastic	FABI2_ORYSJ	39.0	9.7	162.8	3.5	*Oryza sativa* subsp. japonica	Cytoskeleton	− 6.5	− 2.0
41	8413	V-type proton ATPase subunit B 1	VATB1_HORVU	54.0	5.0	1401.8	46.3	*Hordeum vulgare*	Energy processes	− 6.7	− 2.1
42	9006	Oxygen-evolving enhancer protein 1, chloroplastic	PSBO_WHEAT	34.7	9.5	791.1	26.8	*Triticum aestivum*	Photosynthesis	− 32.6	− 3.9
43	3204	Protein disulfide-isomerase like 2–1	PDI21_ARATH	39.5	5.7	63.5	2.8	*Arabidopsis thaliana*	Protein metabolic process	− 6.0	− 2.5

Two DH lines of triticale differ significantly in ME responsiveness (highly recalcitrant DH19 and responsive DH28). Swiss-Prot database was used for protein identification. The results are based on nano LC–MS/MS analyses.

^a^The theoretical molecular weight (MW. kDa) and ^b^ isoelectric point (pI) retrieved from the protein database.

^c^The score and ^d^protein sequence coverage (Score in Flex Analysis software).

^e^Change in abundance was calculated by dividing the mean %vol of a spot in anthers isolated form low temperature treated tillers (3 weeks at 4 °C) to mean %vol of that spot in anthers isolated from AC treated tillers (5.0 µM).

The proteins were grouped according to their way of changes: *the significant up-regulation (≥ 2.0 fold change) in responsive DH28 line and simultaneously down-regulation (≤ − 2.0 fold change) or no significantly change (< │2.0│ fold change) in recalcitrant DH19 line; **the significant down-regulation (≤ − 2.0 fold change) in responsive DH28 line and simultaneously up-regulation (≥ 2.0 fold change) or no significantly change (< │2.0│ fold change) in recalcitrant DH19 line; ***the significant up-regulation (≥ 2.0 fold change) in both DH lines but with higher fold change in DH28 (approx. 1.5); ****the significant down-regulation (≤ − 2.0 fold change) in both DH lines but with higher fold change in DH28 (approx.. 1.5).

To summarize, 5-azacytidine treatments caused significant quantitative changes (prevalently down-regulation) in anther protein profiles, with the highest number of DAPs observed after AC5.0 treatment.

### Changes in anther protein profiles induced by 5.0 µM 5-azacytidine treatment

As the treatment of tillers with AC5.0 improved the effectiveness of ME induction (ELS/100A) in the responsive line DH28, proteins that showed different abundance after this treatment were analyzed with particular interest. Among them, four proteins were significantly up-regulated in DH28, while being down-regulated in DH19 (Table 1). These proteins were involved in: (a) pentose phosphate pathway (ribose-5-phosphate isomerase A, no. 6716), (2) glyoxylate metabolism (putative aconitate hydratase, no. 5904), (3) cell division (cell division control protein 48 homolog A, no. 8903), (4) stress response (heat shock 70 kDa protein 2, no. 9613). The fold change of other proteins associated *inter alia* in carbohydrate metabolism (e.g. xylose isomerase, no. 7512), cell division (cell division control protein 48 homolog D, no. 6902) or protein metabolism (26S proteasome non-ATPase regulatory subunit 7 homolog A, no. 2203) were higher (at least 1.5-fold) in the responsive DH28 line than in line DH19 (Table 1). There were also proteins involved in energy process (ATP synthase subunit alpha, no. 2503) and Calvin cycle (ribulose bisphosphate carboxylase/oxygenase activase A, no. 8202) up-regulated in both DH lines, but with higher fold change in responsive DH28.

Five proteins were significantly down-regulated in anthers of highly responsive DH28 and significantly up-regulated in recalcitrant DH19 (Table 1). Among them, the difference in the expression level of the protein associated with the regulation of photosystem II (oxygen-evolving enhancer protein 2, no. 5007) was the highest (17.7 vs. − 6.7 in DH19 and DH28, respectively, Table 1, Fig. 3a). The majority of identified protein species were down-regulated in anthers of DH28 line, and almost not changed in recalcitrant DH19 (Table 1). They were involved in carbohydrate metabolism, such as pentose phosphate pathway (6-phosphogluconate dehydrogenase, decarboxylating 1, no. 5305) or gluconeogenesis (triosephosphate isomerase, no. 7002). The highest change in expression showed the last mentioned protein (-19.3 *vs* 1.1 in DH28 and DH19, respectively). One of the highest amplitudes in protein expression level was recorded for chlorophyll a-b binding protein 8 (no. 8002)-part of the light-harvesting complex (LHC)-with a 12.8-and 1.6-fold change in DH28 and DH19, respectively (Table 1).

The most pronounced effect was induced by AC5.0 treatment on oxygen-evolving enhancer protein 1 (no. 9006)-a protein necessary for the proper function of photosystem II-whose accumulation in anthers of responsive line DH28 was dramatically reduced (approx. 33-fold). A similar but a markedly weaker effect was observed in line DH19, resulting in an approximately 4-fold reduction in the amount of this protein (Table 1). The same pattern of changes (down-regulation in both DH lines, but with higher fold change in responsive DH line) was observed in protein spots identified as fructose-bisphosphate aldolase (no. 8103) and transketolase (no. 7706)-involved in carbohydrate metabolism, enoyl-[acyl-carrier-protein] reductase [NADH] 2 (no. 7001) and actin-7 (no. 8307)-associated with cytoskeleton, V-type proton ATPase subunit B 1 (no. 8413) a component of vacuolar proton pumps and protein disulfide-isomerase like 2-1 (no. 3204) responsible *inter alia* for proper protein folding (Table 1).

Taken together, proteins that showed different abundance after AC5.0 treatment in the responsive line DH28 were mainly involved in suppression of intensive anabolic processes (e.g. photosynthesis), transition to effective catabolism by mobilization of carbohydrate reserve (e.g. starch and sucrose breakdown) and effective stress-defense (e.g. proper folding during protein biosynthesis).

### Changes in anther protein profiles induced by 10 µM AC treatment

Significant changes were observed in abundance for 17 identified proteins in both DH lines after AC10 treatment (Fig. 3c). As this treatment seems to have some positive effect on green plant regeneration ability in responsive DH28, our attention was focused especially on four DAPs (Table 2). Among them, only one protein was significantly up-regulated: V-type proton ATPase catalytic subunit A (no. 8609), which was associated with energy processes. Three other proteins: two related to carbohydrate metabolism (glyceraldehyde-3-phosphate dehydrogenase 1, cytosolic, no. 0206 and ribose-5-phosphate isomerase A, no. 6716) and one to energy processes (ATP synthase subunit beta, mitochondrial, no. 4302) were down-regulated (Table 2). Its importance seems to be confirmed by the fact that accumulation of these proteins in the anthers of the recalcitrant line DH19 did not changed significantly (no. 8609, no. 0206, no. 4302) or was more dramatically reduced in comparison to DH28 (no. 6716).

**Table 2:** A list of anther proteins, which accumulation significantly changed after microspore embryogenesis (ME) inducing triticale tillers treatment combining low temperature (3 weeks at 4 °C) with the application of 10 µM of 5-azacytidine, DNA demethylating agent.

No.	Spot no.	Protein name	Accession	MW^a^ (kDa)	pI^b^	Protein score^c^	Seq. Cov [%]^d^	Reference organism	Protein function	Fold change^e^	
										DH28	DH19
**Up-regulated in responsive line (DH28) and slightly changed in recalcitrant line (DH19)**											
1	8609	V-type proton ATPase catalytic subunit A	VATA_HORVU	64.1	5.3	468.2	23.4	*Hordeum vulgare*	Energy processes	2.7	1.5
**Down-regulated in responsive line (DH28) and slightly changed in recalcitrant line (DH19)**											
2	206	Glyceraldehyde-3-phosphate dehydrogenase 1. cytosolic	G3PC1_HORVU	36.5	6.8	956.8	31.8	*Hordeum vulgare*	Carbohydrate metabolism	− 2.2	− 1.3
3	6716	Ribose-5-phosphate isomerase A	RPIA_RHOS5	27.0	4.7	45.2	3.8	*Rhodobacter sphaeroides*	Carbohydrate metabolism	− 2.0	− 5.0
4	4302	ATP synthase subunit beta. mitochondrial	ATPBM_ORYSJ	58.9	5.9	282.0	10.9	*Oryza sativa subsp. japonica*	Energy processes	− 2.0	− 1.5

Two DH lines of triticale differ significantly in ME responsiveness (highly recalcitrant DH19 and responsive DH28). Swiss-Prot database was used for protein identification. The results are based on nano LC–MS/MS analyses.

^a^The theoretical molecular weight (MW. kDa) and ^b^ isoelectric point (pI) retrieved from the protein database.

^c^The score and ^d^protein sequence coverage (Score in Flex Analysis software).

^e^Change in abundance was calculated by dividing the mean %vol of a spot in anthers isolated form low temperature treated tillers (3 weeks at 4 °C) to mean %vol of that spot in anthers isolated from AC treated tillers (10 µM).

Collectively, in responsive DH line, AC10 treatment changed the proteins level associated with energy production and cellular homeostasis.

### Expression levels of TaMET2B and ME-associated genes after 5-azacytidine treatments

**Figure 4:**
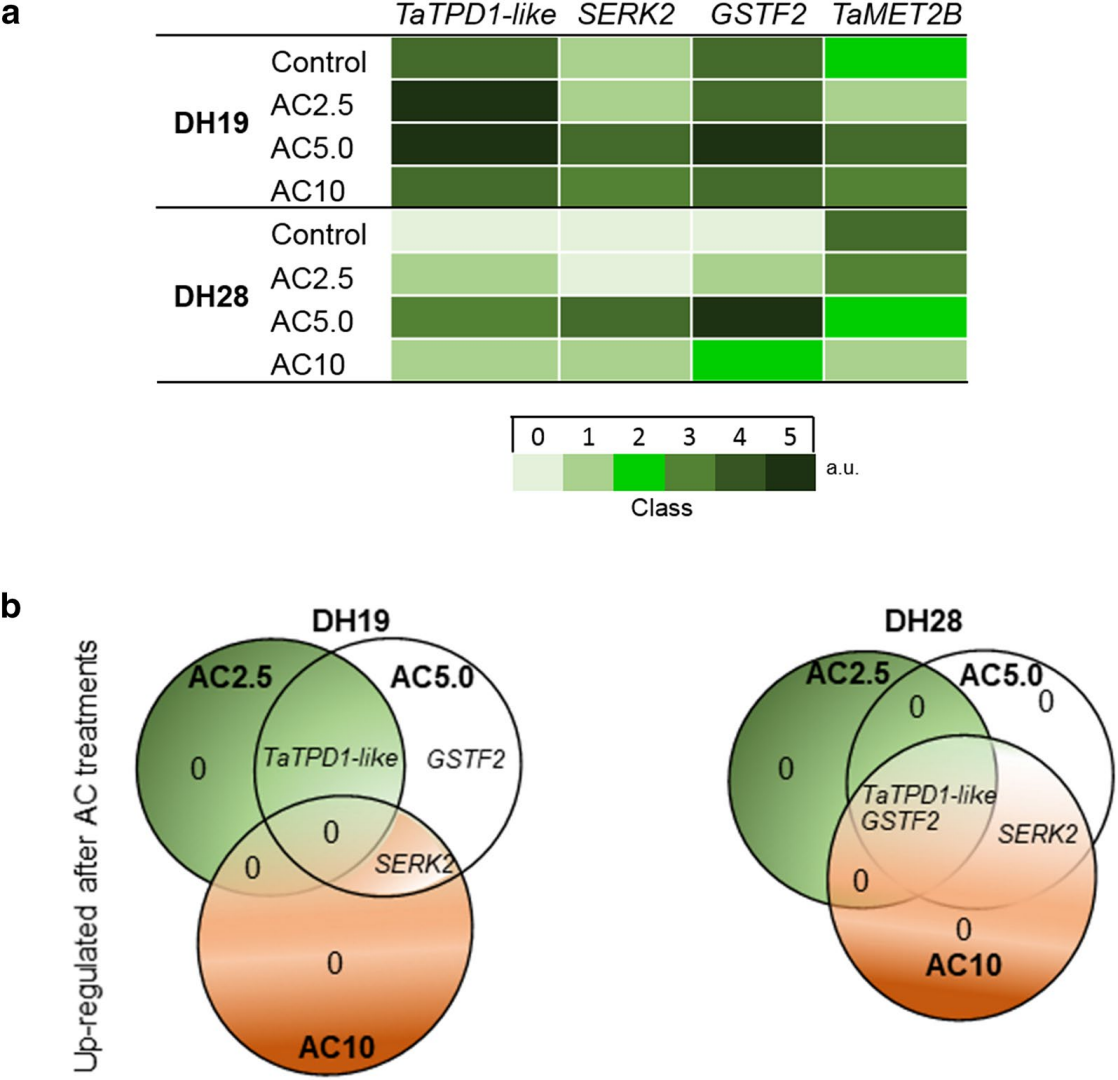
The effect of 5-azacytidine (AC) on the expression of *TaMET2B* and genes associated with microspore embryogenesis induction (*TaTPD1-like*, *SERK2*, *GSTF2)* in the anthers of two doubled haploid (DH) lines of triticale, significantly differed in their responsiveness to microspore embryogenesis (responsive DH28 and recalcitrant DH19). AC was applied at concentrations of 2.5 µM (AC2.5), 5.0 µM (AC5.0) and 10 µM (AC10) during low temperature tillers treatment (3 weeks at 4 °C, control). (**a**) Heat map showing the genes expression. The categorisation (0–4 Classes, when 0 = no signal, Class 4 = max) of the mean intensity expressed in arbitrary units (a.u.). (**b**) Venn diagrams shows the overlap of up-regulated genes after AC treatments in comparison to the control.

The expression of an ortholog of the wheat DNA methyltransferase 2B gene (*TaMET2B)* was higher in anthers isolated after standard tillers treatment (control) in responsive DH28 than in recalcitrant DH19 (Fig. 4a, Supplementary Fig. S3). All AC treatments in DH28 anthers reduced the level of the analyzed transcript, whereas visible down-regulation in DH19 was observed only after AC2.5 treatment. Surprisingly, treatment with AC5.0 and AC10 caused up-regulation of *TaMET2B* (Fig. 4a, Supplementary Fig. S3).

In control anthers, transcripts of three genes (*TaTPD1-like, SERK2* and *GSTF2*) were detected only in DH19 anthers, while none of them was transcribed in the responsive line DH28 (Fig. 4a, Supplementary Fig. S3).

The AC2.5 treatment did not change the levels of *SERK2* and *GSTF2* and slightly up-regulated *TaTPD1-like* expression in DH19 anthers, while it induced the expression of both *TaTPD1-like* and *GSTF2* in the responsive line DH28 (Fig. 4a,b). Whereas, the AC5.0 treatment resulted in the up-regulation of all transcripts in both studied DH lines (Fig. 4a,b). The highest concentration of DNA methylation inhibitor (10 µM) increased the amount of only one transcript (*SERK2*) in DH19 anthers (Fig. 4b). The same treatment up-regulated all transcripts in DH28 anthers; however, their abundance was visibly lower in comparison to the effect exerted by AC5.0 (Fig. 4a,b, Supplementary Fig. S3).

Summarizing the received data, it could be seen that studied DH lines of triticale differ in the expression of *TaMET2B* and in the pattern of changes induced by DNA methyltransferase inhibitor (AC), what seems to be associated with ME potential. High expression of *SERK2*, *GSTF2* and *TaTPD1-like* seems to be important but not the only one element critical for efficient induction of ME.

## Discussion

The precise mechanism of ME and factors important for effective DH production still remain one of the scientific riddles for contemporary researchers. Despite almost half a century of research on this fascinating process, knowledge about it is fragmentary, as the successful ME induction, proper ELS development and its regeneration into a green plant are influenced by many environmental and endogenous factors and their interactions.

Numerous changes occur at different levels during microspores reprogramming, from the overall cell structure, organization and functioning to alterations in the genome, proteome and metabolome^3^. A growing number of reports support the assumption that epigenome regulations also play an important role^25^. Studies in oilseed rape and barley revealed that DNA or histone (H3K9) hypomethylation was beneficial for the earliest stages of ME, whereas pollen development as well as later ME stages were accompanied by a progressive increase in global DNA and histone methylation^23,25,28^.

Many factors important for triticale microspore reprogramming have been identified using a mapping population (‘Saka 3006’ × ‘Modus’), composed of 90 DH lines, or individual DH lines selected from it, highly differentiated in ME responsiveness^6^. Recently, physiological^29–31^, molecular^12,32,33^ and proteomic^16^ studies have been complemented by studies on the epigenetic aspects of ME regulation^26^. Since DNA demethylating agents cause chromatin relaxation and dysregulation of some marker genes associated with ME, our research focused on changes in anther protein profiles and expression levels of selected ME-associated genes with respect to ME efficiency following AC tillers treatment. We used two DH lines (DH19 and DH28) that differed significantly in ME effectiveness and responded in a different manner to AC-induced hypomethylation^26^.

Our earlier study, using the same triticale models, revealed 31 proteins involved in microspore competence for embryogenesis determination, stress response and regulation, and their accumulation was associated with ME induction triggered by low temperature tillers treatment^16^. In the present study, the abundance of 16 of them was also changed, in association with ME induced by low temperature combined with AC5.0 treatment in DH28. Among them, seven proteins associated with carbohydrate metabolism (phosphoglycerate kinase), photosynthesis (oxygen-evolving enhancer protein 1), protein metabolism (protein disulfide-isomerase like 2-1), amino acids biosynthesis (S-adenosylmethionine synthase 3,5-methyltetrahydropteroyltriglutamate-homocysteine methyltransferase 1), energy processes (V-type proton ATPase subunit B 1) and stress response (heat shock 70 kDa protein 3) were down-regulated in DH28 after AC5.0 in opposite to our earlier result, where the up-regulation after ME-inducing treatment in responsive DH lines was observed. It was also shown that chemically induced DNA hypomethylation lowered the abundance of most of identified proteins, what, according to Uváčková et al.^17^, suggests the predominance of catabolic pathways in cell metabolism. Sources of carbon, such as starch, fatty acids and glutamine are broken down in catabolic reactions to generate energy in the form of adenosine triphosphate (ATP), which is used to maintain cell functioning during reprogramming of developmental pathway, following by necessary structural reconstructions and physiological modifications.

### 5-Azacytidine altered the level of proteins involved in carbohydrate metabolism important for effective embryo-like structures formation in responsive DH line of triticale

Increased accumulation of proteins associated with the pentose phosphate pathway (ribose-5-phosphate isomerase A) and Calvin cycle (ribulose bisphosphate carboxylase/oxygenase activase A and ribulose bisphosphate carboxylase large chain), along with down-regulation of proteins like oxygen-evolving enhancer protein 1, 2 and chlorophyll a-b binding protein, observed in genotype DH28 after AC5.0, could be the evidence for the suppression of light-dependent reactions and activation of the light-independent (dark) phase of photosynthesis. This seems to be confirmed by the fact that proteins involved in the chlorophyll and RuBisCO biosynthetic pathways (coproporphyrinogen-III oxidase and RuBisCO large subunit-binding protein subunit beta) were also down-regulated after AC5.0 treatment, which was the most effective in stimulation of ELS formation in DH28.

Previous studies have demonstrated that carbohydrate metabolism is associated with the disruption of male gametophyte development^34^. Carbohydrates also play a similarly important role in somatic embryogenesis, as proteins associated mainly with carbohydrate metabolism differentiated the embryogenic from non-embryogenic callus of maize^35^ and sugarcane^36^.

Our earlier results^16^ indicated a potential role of glyceraldehyde-3-phosphate dehydrogenase (GAPDH) and enolase (ENO) in ME induction. The involvement of these enzymes together with phosphoglucomutase (PGM) in microspore reprogramming confirms the results of the present work. The fold change of all these proteins-associated, *inter alia*, with glycolysis-were higher in the responsive line DH28 in comparison to the recalcitrant line DH19 after the most effective ME-inducing treatment (AC5.0). The accumulation of these enzymes could be related to the higher cell energy demand and cell wall remodeling during the switch of the microspores developmental pathway. Microspores ready for dedifferentiation readjust glucose metabolism by shifting toward anaerobic glycolysis in order to survive in low oxygen conditions.

The highest number of down-regulated proteins was associated with carbohydrate metabolism (e.g. fructose-bisphosphate aldolase (FBA) and UTP-glucose-1-phosphate uridylyltransferase (UGPase). Similarly to our results, Maraschin et al.^10^ reported decreased expression of genes involved in starch biosynthesis, such as UGPase, sucrose synthase 1 (SS1), PGM, UDP-glucose 4-epimerase, glucose-1-phosphate adenylystransferase (AGPase B) and granule-bound starch synthase (GBSS1) in barley microspores induced for embryogenic development. The observed simultaneous up-regulation of genes involved in starch and sucrose breakdown^10^, along with increased accumulation of xylose isomerase in responsive DH28 line, have supported the hypothesis that the repression of starch synthesis is a reaction characteristic for the blockade of gametophytic development during ME induction. It seems that the inhibition of anabolic processes such as gluconeogenesis (down regulation of FBA and triosephosphate isomerase) can save energy for other more important processes for cell survival and ME induction under stress conditions.

Summarizing, suppression of intensive anabolic processes, transition to effective catabolism and mobilization of carbohydrate reserve are essential steps to effective ELS formation in responsive DH line.

### 5-Azacytidine treatment caused changes in the level of proteins involved in stress response and protein metabolism regulation, and enhanced ME induction in responsive DH line of triticale

Another alteration observed in triticale anther proteome after ME induction was associated with protein metabolism: protection of proper folding during protein biosynthesis and effective degradation of dysfunctional or damaged proteins. Among proteins up-regulated in highly responsive DH28 following AC5.0 treatment was heat shock 70 kDa protein 2, which together with disulfide-isomerase (PDI) affect the rate limiting stage in maturation and correct folding of proteins important for cell division and differentiation^37^. Although the PDI-like 2-1 enzyme, which stimulates disulfide bond formation, reduction or isomerization was down-regulated in both DH lines, recalcitrant DH19 was characterized by a visibly higher fold change in the abundance of this protein. It could be explained by its lower tolerance to stress induced by low temperature tiller treatment^29,31^. This hypothesis has confirmed our previous studies, indicating that DH lines of triticale of high embryogenic potential are characterized by a significantly more efficient system of antioxidant defense^7,31^. Furthermore, catabolic metabolism in the recalcitrant line DH19 does not seem to be sufficiently effective as the chloroplast ClpC1 chaperone was strongly down-regulated, while a component of the 26S proteasome (26S proteasome non-ATPase regulatory subunit 7) was only slightly up-regulated in comparison to the responsive line DH28. Both aforementioned proteins are involved in the ATP-dependent degradation of denatured, undesirable proteins in chloroplasts^38^ and cytosol^39^. Maraschin et al.^10^ also observed increased expression of genes involved in protein degradation (26S protease subunit-8, ubiquitin-conjugating enzyme, 20S proteasome subunit alpha-5 and alpha-2) in embryogenic microspores of barley. Other proteins involved in the ubiquitin-26S proteasomal pathway, like cell division control protein 48 homolog A and D, were also more abundant following AC5.0 treatment in the responsive line DH28. Belonging to the AAA-ATPase family, these proteins have a wide range of cellular functions, including endoplasmic reticulum-associated protein degradation, DNA replication and cell proliferation^40^. Su et al.^41^ also identified members of this protein family as involved in the regulation of the early ME stage in *Brassica oleracea*.

Taken together, effective defense against stress induced by low temperature tillers treatment related with proper protein metabolism is another important aspect of effective ME induction.

### 5-Azacyitidine modified the level of proteins associated with cell wall remodeling and cytoskeleton rearrangement that seems to play important role in the embryo-like structures formation in responsive DH line of triticale

Our results indicated that AC5.0 treatment caused the accumulation of beta-galactosidase 1 (BGAL) in both DH lines, although its level was higher in DH28. This enzyme regulating metabolism of galactosyl conjugates during carbohydrate reserve mobilization, cell wall remodeling and turnover of signaling molecules^42^, was also indicated as ME associated in our previous work^16^. The subcellular location of six BGAL proteins from the a1 subfamily was detected in the cell wall of *Arabidopsis thaliana* and their proposed function was associated with cell expansion or stress response^43^. Higher levels of this enzyme could be explained by intensive cell wall remodeling accompanying high mitotic activity in embryogenic cells^44^.

Studies on ME in barley^8^ showed that the expression level of the actin gene (*ACT7*) was positively related to the number of ELS produced in anther cultures. On the contrary, two proteins associated with cytoskeleton organization (actin-7 (ACT7) and formin-like protein 1) were down-regulated in our experiment in the highly responsive line DH28 after AC5.0 treatment. Therefore, it could be assumed that a higher abundance of actin filaments (AF) in winter triticale anthers was not required for high ME effectiveness. Similar conclusions, based on AF depolymerization associated with elevated Ca^2+^ levels, observed in embryogenic microspores of oilseed rape and wheat, were drawn by Gervais et al.^45^ and Castillo et al.^46^.

Collectively, enzymes involved in cell wall remodeling and cytoskeleton organization also appears to be important components of the system that determines the formation of ELS.

### Proteins involved in carbohydrate metabolism and energy processes enhance the capacity of green plants regeneration of responsive DH line of triticale

The effect induced by AC10 was not so pronounced, what could be explained by the cytotoxicity of the applied chemical that depended on the concentration and duration of treatments^47^. A similar effect was observed in Nowicka et al.^26^ after application of 10 and 20 µM demethylating agents. Therefore, when working with DNA methylation inhibitors, like AC or 2′-deoxy-5-azacytidine (DAC), it should be remembered that they are highly toxic and induce many side-effects affecting cell viability and metabolism^48^.

Higher green plants regeneration capacity observed in anther cultures of DH28 after AC10 treatment seems to be associated with the change in energy production and the ability to sustain cellular homeostasis. Up-regulated V-ATPase uses the energy released by ATP hydrolysis to transport protons (H+) across cell membranes to regulate pH in the plant endomembrane system. In addition, V-ATPase plays a role in multiple stress responses including oxidative stress^49^. Control over the level of reactive oxygen species (ROS) generation could be also the aim of down-regulation of ATP synthase subunit beta, mitochondrial and two proteins involved in carbohydrate degradation such as: ribose-5-phosphate isomerase A and glyceraldehyde-3-phosphate dehydrogenase 1.

It could be assumed that proper energy management and carbohydrate metabolism results in higher efficiency of green plant regeneration.

### 5-Zacitidine treatment altered ME-associated gene expression profiles

In addition to significant changes in anther protein profiles, some differences in transcript levels of genes associated with ME and the gene encoding methyltransferase 1, were also revealed as following the treatments with DNA methylation inhibitor.

As expected, transcript level of *TaMET2B* in the responsive line DH28 was decreasing with increasing AC concentration. In contrast, the pattern of transcriptional activity of the gene encoding DNA methylase was not directly associated with AC concentration in the recalcitrant line DH19. This could be explained by the fact that global DNA methylation level is not directly linked to the activity of DNA methylases^50^ and may be a consequence of the complexity of interactions controlling the balance between DNA replication, de novo/maintenance DNA methylation and demethylation^51^.

Previously, Nowicka et al.^26^ showed that low temperature caused differential changes in the expression of genes, including *GSTF2, TaTPD1-like* and *SERK2,* in microspores of recalcitrant DH19 and responsive DH28 triticale DH lines.

Up-regulation of *GSTF2* during the first stages of ME in different species has been described^9,12,26^. Its role is probably related to cell protection against the harmful effects of ROS generation, enhancement of microspores viability and reprogramming efficiency^31,52^. Similarly, the expression of *SERK2*, a member of the receptor-like kinase subfamily, is known to be associated with early stages of embryogenesis, in the zygotic, somatic^53^ and gametic form of this process^54^. The expression profile of *SERK2* appears to be specific for plant species, culture type and/or stress treatment^12,13,26,54^ and also seems to be involved in the response to (a)biotic stress^55^. AC treatment also altered transcript level of the *TaTPD1-like* gene, which participates in the early ME signaling pathways. Expression of this gene was induced in wheat zygotic embryos^56^ and in wheat anthers on the 5^th^ day of in vitro culture^13^. Moreover, an increased transcript level of this gene was observed after treatment with histone deacetylase inhibitor (trichostatin A, TSA) on the 3^rd^ day of culture in two bread wheat cultivars with different androgenic response^46^. Similarly, its up-regulation was observed in triticale anthers after 4 days of in vitro culture^12^, and in triticale microspores after ME-inducing low temperature treatment^26^. In this experiment, the combination of low temperature and treatment with 5.0 µM AC up-regulated the expression of all studied genes in both DH lines of triticale.

## Summary and conclusions

The application of AC at 5–10 µM concentration range during ME-inducing low temperature tillers treatment decreased the expression of *TaMET2B* gene what was associated with increased efficiency of ELS formation (AC5.0) or green plant regeneration capacity (AC10) in anther cultures of highly responsive triticale line DH28. Stimulation of ELS development induced by AC5.0, enhanced the expression of *GSTF2*, *TaTPD1-like* and *SERK2* genes involved in ME regulation. The AC2.5 and AC5.0 treatments resulted also in some green plant regeneration in recalcitrant line DH19, however the efficiency of the process was so low that, none of applied treatments could be consider as having significant positive influence on ME effectiveness in this recalcitrant line. The differences in ME effectiveness between the studied DH lines were also reflected in the diversity of alterations induced in triticale anther protein profiles by AC treatments. The most important changes associated with more effective ME induction concerned.

1. Suppression of intensive anabolic processes: mainly photosynthesis-by down-regulation of genes controlling chlorophyll biosynthesis, RuBisCO and light-depended reactions (chlorophyll a–b binding protein, coproporphyrinogen-III oxidase, RuBisCO large subunit-binding protein subunit beta, oxygen-evolving enhancer protein 1).
2. Transition to effective catabolism and mobilization of carbohydrate reserve of genes coding enzymes involved in starch and sucrose breakdown, and glycolysis (such as glyceraldehyde-3-phosphate dehydrogenase, enolase, phosphoglucomutase) to meet intense energy requirements of reprogramming microspores.
3. Initiation of effective defense against stress induced by low temperature tillers treatment-securing proper folding of de novo synthetized proteins (heat shock 70 kDa), effective degradation of dysfunctional or damaged proteins (26S proteasome non-ATPase regulatory subunit 7).

The presented results have confirmed that DNA demethylating agents possess the potential to increase ME effectiveness also in triticale. However, its wider implementation in DH technology requires further in-depth research and procedure optimization, especially for more recalcitrant genotypes.

## Methods

### Plant material

Two DH lines (DH19, DH28) of winter hexaploid triticale with different ME effectiveness were chosen for the study. Both lines were selected from the mapping population of 90 DH lines ‘Saka 3006’ × ‘Modus’^57^ obtained from the State Plant Breeding Institute at the Hohenheim University (Stuttgart, German), complies with relevant institutional, national, and international guidelines and legislation.

Based on the data collected from three separate phenotyping, DH28 was identified as highly responsive to ME induction, while DH19 was identified as a recalcitrant model, which was confirmed later in several experiments^29,30,32,33^. The procedures of seeds germination, vernalisation and plant growth conditions were described by Żur et al.^29^.

### ME-inducing stress treatment

Tillers were harvested when most of microspores were at mid- to late-uninucleate development stage, then wrapped in plastic bags, placed in jars containing Hoagland’s salt solution (HSS, according to Wędzony^58^) and stored at 4 °C in the dark for 3 weeks. AC (Sigma-Aldrich; A2385) was added to HSS at a concentration of 2.5, 5.0 and 10 μM for the last 4 days before anther isolation. The solution was refreshed three times every 24 h. After control (low temperature) and combined (low temperature with AC) tillers treatments, the anthers were isolated and transferred to in vitro culture or collected for proteomic and molecular analyses (immediately frozen in liquid nitrogen and stored at  -80 °C).

### Anther culture protocol

The protocol for anther culture was described earlier by Wędzony^58^ and applied here as in the studies of Krzewska et al.^32^ and Żur et al.^59^ with some modifications. Briefly, aseptically excised anthers were placed in 60 × 15 mm Petri dishes (100 anthers from spike per dish) containing the induction medium C17^60^, modified according to Krzewska et al.^32^. The cultures were placed in the dark at 28 ± 1 °C. The produced ELS were transferred successively starting from the 6th week of culture at 3-week intervals. The ELS were placed in 90 × 20 mm Petri dishes (30 ELS per dish) with the regeneration medium 190-2R^61^, containing 30 mg/l sucrose, 0.5 mg/l kinetin, 0.5 mg/l NAA and 0.6% agar, pH 6.0. The regeneration phase took place at 26 °C, in the light (at about 30 µmol/m^2^/s^1^ for the first week, then increased to 80–100 µmol/m^2^/s^1^) with 16/8 h (day/night) photoperiod.

### Evaluation of microspore embryogenesis effectiveness in anther cultures

Three parameters were selected in order to describe the ME effectiveness: (1) ELS/100A-the number of ELS per 100 anthers (A), (2) GR/100A-the number of green regenerants per 100 anthers, (3) AR/100A-the number of albino regenerants per 100 anthers.

Each dish containing 100 anthers collected from one spike was considered be a replicate. Mean values were calculated from at least three replicates.

Data were examined by two-way analysis of variance (ANOVA), while post hoc comparison was conducted using Duncan’s multiple range test (*p* ≤ 0.05). Statistical analyses were performed using STATISTICA v. 13.1 (Stat Soft Inc., USA, 2016) packages.

### Protein extraction

Total soluble proteins were extracted from anthers isolated from low temperature-treated tillers (control), as well as from anthers isolated from tillers treated with low temperature in the presence of different AC concentrations (2.5, 5.0 and 10 µM). The protein isolation protocol was performed according to Klubicova et al.^62^. The collected anthers (approx. 1 g fresh weight; FW) were ground to a fine powder in liquid nitrogen using a mortar and pestle. After adding 10 ml of phenol-based extraction buffer and centrifugation (5000×*g* for 15 min at 4 °C), proteins were precipitated from the phenol phase using 0.1 M ammonium acetate in methanol and collected by centrifugation (5000×*g* for 10 min at 4 °C). Subsequently, protein pellet was washed twice with 0.1 M ammonium acetate in methanol, next twice with 80% acetone and once with 70% ethanol. The pellet was finally dissolved in 200 µl of isoelectric focusing (IEF) sample solution (8 M urea, 2 M thiourea, 2% (w/v) CHAPS, 2% (v/v) Triton X-100, 50 mM DTT). Protein concentration was determined with the 2-D Quant Kit (GE Healthcare) using bovine serum albumin as a standard. Protein aliquots of (500 µg) were stored at -80 °C until further analysis.

### Two-dimensional gel electrophoresis

Protein aliquots were defrosted, mixed with 3.2 µl of ampholytes (pH 5–8), adjusted to a final volume of 315 µl with IEF sample solution and subsequently loaded onto immobilized pH gradient (IPG) strips (pH 5–8, 17 cm, linear gradient, Bio-Rad) in an isoelectric focusing unit (Protean IEF Cell, Bio-Rad). IEF running conditions were previously described by Klubicova et al.^62^. After the first dimension IPG strips were equilibrated for 15 min in 5 ml of SDS equilibration buffer (1.5 M Tris–HCl pH 6.8, 6 M urea, 30% (v/v) glycerol, 5% (w/v) SDS) with 2% (w/v) DTT, followed by 15 min with the same buffer but containing 2.5% (w/v) iodoacetamide (IAA) instead of DTT. The strips were then transferred to 12% SDS–polyacrylamide gels and overlayed with 0.5% (w/v) agarose in SDS running buffer with bromophenol blue addition as a tracking dye. The second dimension was conducted at 10 mA/gel until the dye front reached the bottom of the gel (approx. 16 h) using Protean II xi Cell (Bio-Rad).

Gels (in triplicates) were stained overnight in colloidal Coomassie Brilliant Blue (CBB G-250), digitalized using the ChemiDoc MP System (Bio-Rad) and documented by the Image Lab Program v. 4.1 (Bio-Rad). Image analyses (normalization, spot matching, expression analyses and statistics) were performed using the PDQuest 8.0 software (Bio-Rad). Firstly, gels images were inverted, centralized and cropped according to the same anchor spot, subsequently the correlation coefficient between replicates was checked. The Master Gel was selected automatically and used for all bioinformatic analyses. Relative spot intensities were normalized according to total density in the gel images. One-way ANOVA statistical analysis was performed at 95% significance level to determine which protein species abundance differed between the samples collected from control and AC-treated tillers. On the basis of the above calculations, spots showing a statistically significant (*p* ≤ 0.05) increase in abundance (≥ 2-fold) after at least one of the AC treatments were selected and manually picked for digestion and identification. Protein altered in the same manner meant that this protein was up/down-regulated in both DH lines after at least one of the AC treatments (2.5 µM, 5.0 µM or 10 µM).

### Mass spectrometry-based protein identification—nano LC–MS/MS analyses

Protein bands after SDS-PAGE were prepared for mass spectrometry analysis according to a previously published protocol^63^. Briefly, the excised gel pieces were destained in 100 mM ammonium bicarbonate and washed three times with 50% (v/v) acetonitrile to remove CBB. The gel pieces were dried and treated with 50 mM DTT and 100 mM IAA for protein reduction and carbamidomethylation, respectively. Protein digestion was conducted using 10 ng/μl trypsin Gold solution at 37 °C overnight. Digestion products were collected and combined with subsequent fractions. Further peptide extraction was performed in acidic conditions by incubating twice in 50% acetonitrile with 5% formic acid. Lastly, the gel pieces were dehydrated in anhydrous acetonitrile. Combined solutions from one sample were dried in a SpeedVac and dissolved in 20 μl 0.1% formic acid and analyzed by liquid chromatography with mass spectrometry detection.

Nano LC–MS/MS analyses were performed using the Easy-nLC II nanocapillary chromatography system (Bruker Daltonics, Bremen, Germany) as published earlier^64^. Peptides separation was performed using 3-μm Biosphere C18 columns (length-10 cm, ID-75 μm, particle size-3 μm, Nanoseparations, Nieuwkoop, Netherlands). The gradient was formed using two mobile phases; Phase A: 0.1% formic acid in water and Phase B: 0.1% formic acid in acetonitrile at a total flow rate equal to 300 nl/min. The system was controlled by the Hystar software (Bruker Daltonics, Bremen, Germany). Phase B was ran through a 2–45% gradient for 30 min., followed by 90% for 10 min., and again reduced to 2% for 60 min. for column equilibration. Fractions eluted from the column were directly deposited with a matrix on a MALDI target plate using a Proteineer fc II sample collector (Bruker Daltonics, Bremen, Germany). Fifteen-second fractions were collected, 96 fractions per sample in total, and subsequently spotted onto a 384 MALDI target plate. α-Cyano-4-hydroxycinnamic acid was used as a MALDI matrix. Mass spectrometry analyses were performed on Ultraflextreme (Bruker Daltonics, Bremen, Germany) using a positive ion mode.

The acquired mass spectra and fragment mass spectra were analyzed using the Flex Analysis software (Bruker Daltonics, Bremen, Germany) and processed using the Mascot algorithm (Matrix Science) against the Swiss-Prot database. Search parameters were set as follows: taxonomy-all entries, fixed modifications-carbamidomethyl, variable modifications-methionine dioxidation, 1 missed cleavage allowed, peptide charge -+ 1, mass tolerance-25 ppm for precursor mass. Proteins with a mascot score higher than 30, and false positives with *p* ≤ 0.05 were considered identified. The biological function of the identified proteins was assigned using the UniProt database.

### RNA isolation and RT-PCR analyses

Total RNA was isolated from anthers following standard ME induction treatment (low temperature), and after low temperature treatment combined with AC application using the RNease Plant Mini Kit (Qiagen); RNA was purified from DNA using DNase I, quantified spectrophotometrically (BioSpec-nano, Shimadzu) and stored at  -80 °C until use. The RNA purity parameter (OD260/280) was about 2. The Maxima H Minus First Strand cDNA Synthesis Kit (Thermo Fisher Scientific) was used to prepare cDNA from 1 μg of RNA, according to the manufacturer’s instructions. Primers used in this study are listed in Supplementary Table S5. *TaMET2B* ortholog was selected based on Thomas et al.^65^ and genes associated with ME from Żur et al.^12^. The expression profiles of genes previously identified as being associated with the embryogenic potential of microspores: *TAPETUM DETERMINANT1 (TaTPD1-like), SOMATIC EMBRYOGENESIS RECEPTOR KINASE 2 (SERK2)* and *GLUTATHIONE S-TRANSFERASE* (*GSTF2*) were analyzed.

The RT-PCR profile included an initial denaturation step at 95 °C for 5 min, 35 cycles of denaturation at 95 °C for 30 s, annealing at 62 °C for 60 s, extension for 72 °C at 60 s, and final extension at 72 °C 5 for min. RT-PCR products underwent electrophoretic separation in 2% agarose gel with Midori Green staining (5 µl for 100 ml of gel) (Genetics). The visualization of the separated products was carried out using a ChemiDoc MP System #1708280 transilluminator (Bio-Rad).

## Supplementary Information

Supplementary Information.
