# Supplementary material for: Comparative proteomic analysis provides new insights into regulation of microspore embryogenesis induction in winter triticale (× Triticosecale Wittm.) after 5-azacytidine treatment
Source: Sci Rep. 2021 Nov 15;11:22215. doi: 10.1038/s41598-021-01671-y (PMC8593058; doi:10.1038/s41598-021-01671-y)
Supplement: Supplementary file 1 — Supplementary Information. [file 41598_2021_1671_MOESM1_ESM.pdf]

Supplementary information

Supplementary Table S1. The effect of tested variables on embryo-like structures formation in anther cultures of two winter triticale DH lines, after 5-azacytidine tiller treatment.

Sources of variation	MS	p
**(1) Donor plant genotype**	5859	***
**(2) Tiller treatment**	331	***
**(1) × (2)**	421	***

The sources of variance were as follows: two DH lines, three treatments, and interaction between DH line and treatment MS mean square, p probability, *** $p \leq 0.001$

Supplementary Table S2. The total protein yield obtained from anthers of two winter triticale DH lines after various types of tiller treatments and the extraction based on phenol protocol . Data are the means from 3 biological replicates ± SE.

DH line	Treatment	Anthers FW [g]	Protein content [µg/g FW]
**DH19**	**Control**	3.9±0.17	893.1±181[ab]
	**AC2.5**	1.4±0.06	2096.4±526[bc]
	**AC5.0**	2.2±0.16	1507.7±423[ab]
	**AC10**	2.0±0.04	667.6±8.5[a]
**DH28**	**Control**	4.3±0.19	987.2±339[ab]
	**AC2.5**	0.7±0.03	2196.3±652[c]
	**AC5.0**	2.3±0.02	1031.0±63[ab]
	**AC10**	2.8±0.06	479.1±84[a]

DH19 – recalcitrant DH line, DH28 – responsive DH line, Control – anthers isolated from tillers treated with low temperature (3 weeks at 4 °C), AC2.5, AC5.0, AC10 – anthers isolated from tillers treated with low temperature and 5-azacytidine at concentration of 2.5 µM, 5.0 µM and 10 µM respectively, FW – fresh weight of anthers.

Supplementary Figure S1. Electrophoregrams of triticale anther protein map after low temperature (3 weeks at 4 °C, control) and 5-azacytidine tiller treatments (2.5, 5.0 and 10 µM AC) in two winter triticale DH lines significantly differed in their responsiveness to microspore embryogenesis (ME). Electrophoregrams of recalcitrant line DH19 (a) and responsive line DH28 (b). The identified proteins were marked in the different electrophoregrams to better visualization of each protein spot. *Circles* indicate the position of identified differentially abundant proteins. Spots are described in Supplementary Table S4.
a – DH19, **b** – DH28. Figure presented full-length gels.

a

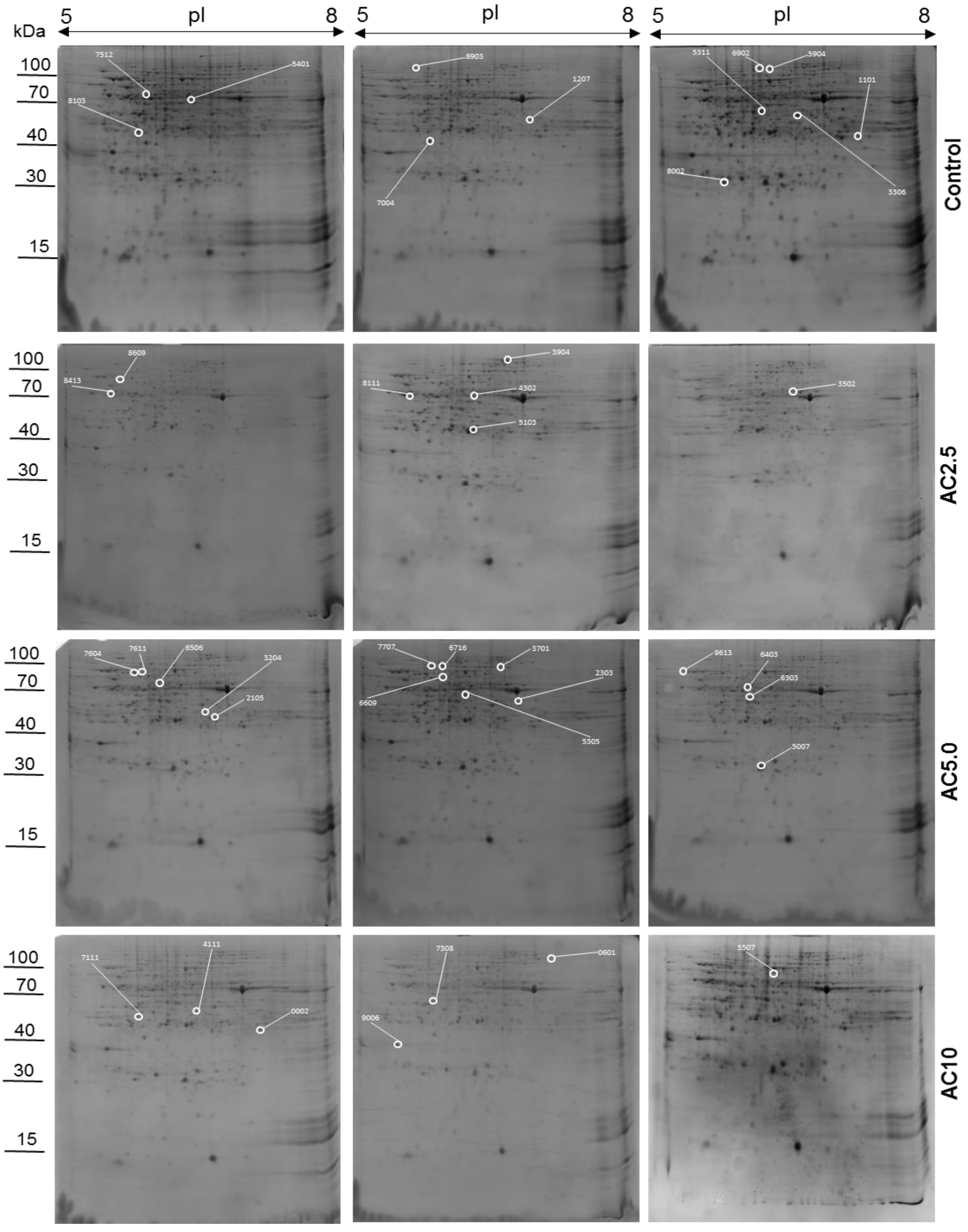

b

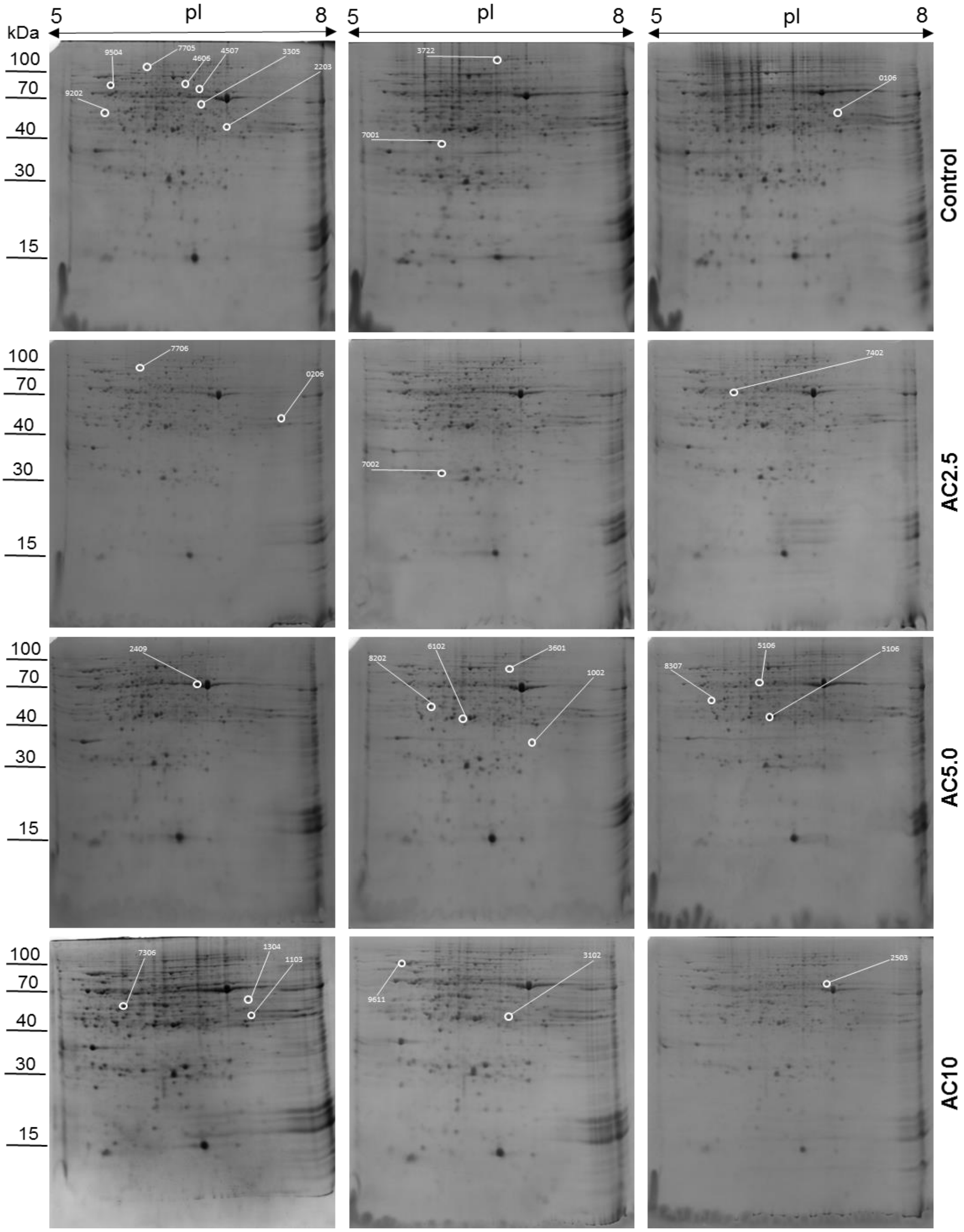

Supplementary Table S3. The effect of tested variables on the number of differentially abundant proteins (DAPs) identified in triticale anthers isolated from tillers after 5-azacytidine treatments.

Sources of variation	F	p
**(1) Donor plant genotype**	23.5	***
**(2) Tiller treatment**	200.4	***
**(1) × (2)**	2.3	NS

The sources of variance for the number of proteins were as follows: two DH lines, three tiller treatments, and interaction between DH line and treatment, F value calculated as variance of a variable or interaction between variables divided by error variance of the experiment, p – probability, *** $p \leq 0.001$, NS – not significant

Supplementary Table S4. List of all proteins identified in the anthers of two DH lines of winter triticale associated with stress treatment of tillers used for microspore embryogenesis (ME) induction. ME was induced by low temperature (3 weeks at 4°C) or low temperature combined with the application of 2.5, 5.0 or 10 μM of 5-azacytidine, DNA demethylating agent. Swiss-Prot database was used for protein identification. The results are based on nano LC-MS/MS analyses.

No.	Spot no.	Protein name	Accession	MW[a] (kDa)	pI[b]	Protein Score[c]	Seq. Cov[d] [%]	Reference organism	Protein function
1	2105	3-isopropylmalate dehydrogenase. chloroplastic	LEU3_SOLTU	39.7	9.2	147.5	7.3	*Solanum tuberosum*	amino acids biosynthesis
2	7707	Acetolactate synthase 1. chloroplastic	ILVB1_MAIZE	68.9	6.8	57.1	3.4	*Zea mays*	amino acids biosynthesis
3	7111	Glutamine synthetase cytosolic isozyme 1-3	GLN13_ARATH	38.6	5.7	100.9	10.2	*Arabidopsis thaliana*	amino acids biosynthesis
4	5311	**S-adenosylmethionine synthase 3**	METK3_HORVU	42.7	5.4	198.7	7.6	*Hordeum vulgare*	amino acids biosynthesis
5	4507	Betaine aldehyde dehydrogenase	BADH_HORVU	54.3	5.8	129.9	5.9	*Hordeum vulgare*	amino acids biosynthesis
6	3701	**5-methyltetrahydropteroyltriglutamate-homocysteine methyltransferase 1**	METE1_ORYSJ	84.5	5.9	737.2	15.7	*Oryza sativa subsp. japonica*	amino acids biosynthesis
7	6506	Ketol-acid reductoisomerase. chloroplastic	ILV5_ORYSJ	62.3	6.0	179.7	5.4	*Oryza sativa subsp. japonica*	amino acids biosynthesis
8	6303	Adenylosuccinate synthetase. chloroplastic	PURA_WHEAT	50.9	5.9	44.3	4.0	*Triticum aestivum*	aromatic compounds metabolism
9	6102	Coproporphyrinogen-III oxidase 1. chloroplastic	HEM61_ARATH	43.8	6.2	176.9	5.7	*Arabidopsis thaliana*	aromatic compounds metabolism
10	6716	Ribose-5-phosphate isomerase A	RPIA_RHOS5	27.0	4.7	45.2	3.8	*Rhodobacter sphaeroides*	carbohydrate metabolism
11	7512	**Xylose isomerase**	XYLA_HORVU	53.6	5.2	460.2	19.0	*Hordeum vulgare*	carbohydrate metabolism
12	3102	**Glyceraldehyde-3-phosphate dehydrogenase 2. cytosolic**	G3PC2_HORVU	36.5	6.8	111.0	9.2	*Hordeum vulgare*	carbohydrate metabolism
13	3904	**Beta-galactosidase 1**	BGAL1_ORYSJ	91.7	5.7	140.5	4.4	*Oryza sativa subsp. japonica*	carbohydrate metabolism

14	5904	**Putative aconitate hydratase. cytoplasmic**	ACOC_ORYSJ	98.0	5.6	89.6	1.1	*Oryza sativa subsp. japonica*	carbohydrate metabolism
15	7402	**Enolase**	ENO_ORYSJ	47.9	5.3	361.0	17.7	*Oryza sativa subsp. japonica*	carbohydrate metabolism
16	0206	**Glyceraldehyde-3-phosphate dehydrogenase 1. cytosolic**	G3PC1_HORVU	36.5	6.8	956.8	31.8	*Hordeum vulgare*	carbohydrate metabolism
17	5507	Phosphoglucomutase. cytoplasmic	PGMC_BROIN	62.6	5.2	68.1	2.6	*Bromus inermis*	carbohydrate metabolism
18	7002	Triosephosphate isomerase. cytosolic	TPIS_HORVU	26.7	5.3	603.2	30.4	*Hordeum vulgare*	carbohydrate metabolism
19	1103	Fructose-bisphosphate aldolase cytoplasmic isozyme	ALF_ORYSJ	36.7	9.0	239.3	8.9	*Oryza sativa subsp. japonica*	carbohydrate metabolism
20	7706	Transketolase. chloroplastic	TKTC_MAIZE	72.9	5.4	72.6	1.9	*Zea mays*	carbohydrate metabolism
21	5103	Malate dehydrogenase. cytoplasmic	MDHC_MESCR	35.5	6.0	389.3	12.0	*Oryza sativa subsp. japonica*	carbohydrate metabolism
22	1304	Citrate synthase 5. mitochondrial	CISY5_ARATH	51.7	6.2	203.8	6.7	*Arabidopsis thaliana*	carbohydrate metabolism
23	8103	Fructose-bisphosphate aldolase. chloroplastic	ALFC_ORYSJ	42.0	6.5	490.9	22.2	*Oryza sativa subsp. japonica*	carbohydrate metabolism
24	9202	**Phosphoglycerate kinase, chloroplastic**	PGKH_WHEAT	49.8	6.7	577.8	20.0	*Triticum aestivum*	carbohydrate metabolism
25	4606	Succinate dehydrogenase [ubiquinone] flavoprotein subunit 1. mitochondrial	SDHA1_ARATH	69.6	5.8	223.4	7.6	*Arabidopsis thaliana*	carbohydrate metabolism
26	6403	Inositol-3-phosphate synthase	INO1_HORVU	56.1	5.4	449.5	15.9	*Hordeum vulgare*	carbohydrate metabolism
27	5305	6-phosphogluconate dehydrogenase. decarboxylating 1	6PGD1_ORYSJ	52.7	5.8	229.7	6.5	*Oryza sativa subsp. japonica*	carbohydrate metabolism
28	0106	Formate dehydrogenase. mitochondrial	FDH_HORVU	41.5	7.1	235.0	11.9	*Hordeum vulgare*	carbohydrate metabolism
29	8111	UTP--glucose-1-phosphate uridylyltransferase	UGPA_HORVU	51.6	5.1	965.6	30.2	*Hordeum vulgare*	carbohydrate metabolism
30	1207	**Isocitrate dehydrogenase [NAD] regulatory subunit 3. mitochondrial**	IDH3_ARATH	39.9	7.8	181.2	12.2	*Arabidopsis thaliana*	carbohydrate metabolism
31	6902	Cell division control protein 48 homolog D	CD48D_ARATH	90.3	4.9	186.7	5.2	*Arabidopsis thaliana*	cell division

32	8903	Cell division control protein 48 homolog A	CD48A_ARATH	89.3	5.0	122.3	5.7	*Arabidopsis thaliana*	cell division
33	7001	Enoyl-[acyl-carrier-protein] reductase [NADH] 2. chloroplastic	FABI2_ORYSJ	39.0	9.7	162.8	3.5	*Oryza sativa subsp. japonica*	cytoskeleton
34	8307	Actin-7	ACT7_ARATH	41.7	5.2	442.4	30.2	*Arabidopsis thaliana*	cytoskeleton
35	5106	Formin-like protein 1	FH1_ARATH	115.1	9.3	50.4	1.5	*Arabidopsis thaliana*	cytoskeleton
36	7306	Actin-104 (Fragment)	ACT7_TOBAC	37.1	5.5	130.9	4.8	*Nicotiana tabacum*	cytoskeleton
37	6609	DNA repair and recombination protein RadB	RADB_THEVO	26.0	8.9	45.9	2.6	*Thermoplasma volcanium*	DNA repair
38	2503	ATP synthase subunit alpha. chloroplastic	ATPA_WHEAT	55.3	6.1	279.3	11.7	*Triticum aestivum*	energy processes
39	8413	**V-type proton ATPase subunit B 1**	VATB1_HORVU	54.0	5.0	1401.8	46.3	*Hordeum vulgare*	energy processes
40	5508	ATP synthase subunit alpha. mitochondrial	ATPAM_MAIZE	55.1	5.8	232.2	15.2	*Zea mays*	energy processes
41	4302	ATP synthase subunit beta. mitochondrial	ATPBM_ORYSJ	58.9	5.9	282.0	10.9	*Oryza sativa subsp. japonica*	energy processes
42	8609	V-type proton ATPase catalytic subunit A	VATA_HORVU	64.1	5.3	468.2	23.4	*Hordeum vulgare*	energy processes
43	3722	Linoleate 9S-lipoxygenase 1	LOX1_HORVU	96.3	5.7	262.6	4.8	*Hordeum vulgare*	fatty acids metabolism
44	2303	3-oxoacyl-[acyl-carrier-protein] synthase I. chloroplastic	KASC1_HORVU	49.0	7.1	83.6	4.5	*Hordeum vulgare*	fatty acids metabolism
45	1101	Malate dehydrogenase. glyoxysomal	MDHG1_ARATH	37.3	9.2	42.7	3.1	*Arabidopsis thaliana*	fatty acids metabolism
46	8202	Ribulose bisphosphate carboxylase/oxygenase activase A. chloroplastic	RCAA_HORVU	51.0	8.9	627.4	17.2	*Hordeum vulgare*	photosynthesis
47	2409	**Ribulose bisphosphate carboxylase large chain**	RBL_WHEAT	52.8	6.2	910.3	19.3	*Triticum aestivum*	photosynthesis
48	9006	**Oxygen-evolving enhancer protein 1. chloroplastic**	PSBO_WHEAT	34.7	9.5	791.1	26.8	*Triticum aestivum*	photosynthesis
49	8002	Chlorophyll a-b binding protein 8. chloroplastic	CB13_SOLLC	24.2	0.0	130.3	5.5	*Solanum lycopersicum*	photosynthesis
50	5007	Oxygen-evolving enhancer protein 2. chloroplastic	PSBP_WHEAT	27.3	9.5	855.9	44.6	*Triticum aestivum*	photosynthesis

51	2203	26S proteasome non-ATPase regulatory subunit 7 homolog A	PSD7A_ARATH	34.7	6.0	201.1	12.7	*Arabidopsis thaliana*	protein metabolic process
52	0002	Probable inactive methyltransferase	METL_ORYSJ	40.0	5.6	60.2	5.1	*Oryza sativa subsp. japonica*	protein metabolic process
53	3204	Protein disulfide-isomerase like 2-1	PDI21_ARATH	39.5	5.7	63.5	2.8	*Arabidopsis thaliana*	protein metabolic process
54	1002	Putative F-box/FBD/LRR-repeat protein At4g26350	FDL28_ARATH	49.6	9.7	40.4	2.1	*Arabidopsis thaliana*	protein metabolic process
55	3305	26S protease regulatory subunit 7	PRS7_ORYSJ	47.7	5.9	194.2	11.0	*Oryza sativa subsp. japonica*	protein metabolic process
56	9504	RuBisCO large subunit-binding protein subunit beta. chloroplastic	RUBB_SECCE	53.4	4.7	530.4	19.2	*Secale cereale*	protein metabolic process
57	7705	Chaperone protein ClpC1. chloroplastic	CLPC1_ORYSJ	101.7	6.1	452.7	12.0	*Oryza sativa subsp. japonica*	protein metabolic process
58	7611	70 kDa peptidyl-prolyl isomerase	FKB70_WHEAT	62.0	5.2	460.9	21.6	*Triticum aestivum*	protein synthesis
59	7308	**Elongation factor Tu. chloroplastic**	EFTU_PEA	53.0	6.7	166.4	5.7	*Pisum sativum*	protein synthesis
60	4111	Elongation factor Tu. mitochondrial	EFTM_ARATH	49.4	6.3	385.0	9.7	*Arabidopsis thaliana*	protein synthesis
61	3502	Oleosin GRP-17	GRP17_ARATH	53.2	10.9	90.0	5.3	*Arabidopsis thaliana*	reproduction sexual
62	9613	**Heat shock 70 kDa protein 2**	HSP72_USTMA	70.3	4.9	282.7	9.5	*Ustilago maydis*	stress response
63	3601	Hsp70-Hsp90 organizing protein 1	HSOP1_SOYBN	64.1	5.4	74.5	2.6	*Glycine max*	stress response
64	7604	Heat shock 70 kDa protein 10. mitochondrial	HSP7J_ARATH	72.9	5.5	446.1	6.2	*Arabidopsis thaliana*	stress response
65	7004	Lactoylglutathione lyase	LGUL_ORYSJ	32.5	5.4	326.8	11.3	*Oryza sativa subsp. Japonica*	stress response
66	9611	**Heat shock 70 kDa protein 3**	HSP7C_ARATH	71.1	4.8	1169.7	25.4	*Arabidopsis thaliana*	stress response
67	3306	Cytosolic isocitrate dehydrogenase [NADP]	ICDHC_ARATH	45.7	6.1	548.5	19.0	*Arabidopsis thaliana*	stress response
68	601	Protein TOC75. chloroplastic	TOC75_ORYSJ	87.6	9.2	211.8	6.7	*Oryza sativa subsp. japonica*	transport
69	5401	Guanosine nucleotide diphosphate dissociation inhibitor At5g09550	GDI_ARATH	49.5	5.0	72.6	8.3	*Arabidopsis thaliana*	transport

[a]The theoretical molecular weight (MW. kDa) and [b] isoelectric point (pI) retrieved from the protein database
[c]The score and [d]protein sequence coverage (Score in Flex Analysis software)
The protein name indicated with **bold** – the proteins previously identified as associated with ME[16].

Supplementary Figure S2. Diagrams showing functional classification of identified differentially abundant proteins in both studied triticale DH lines after AC treatments.

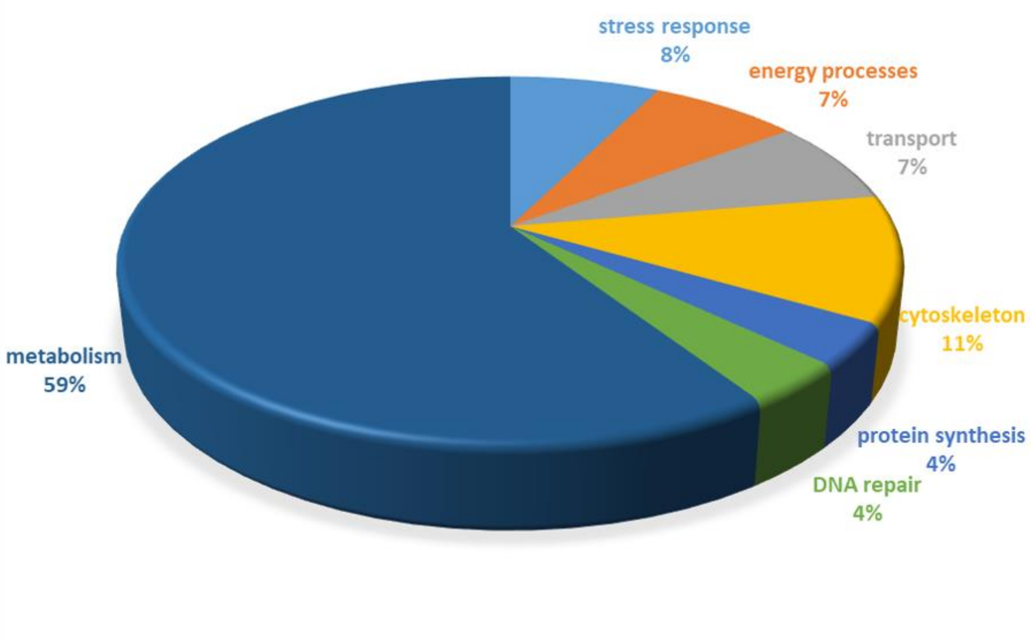

Supplementary Figure S3. The effect of low temperature (3 weeks at 4 °C, Control) and 5-azacytidine (AC) tiller treatments on the expression level of selected genes (*TaMET2B, TaTPD1-like, SERK2, GSTF2*) associated with microspore embryogenesis (ME) induction in the anthers of two winter triticale doubled haploid (DH) lines (responsive DH28 and recalcitrant DH19). AC was applied at concentrations of 2.5 µM (AC2.5), 5.0 µM (AC5.0) and 10 µM (AC10). *18S rDNA* was used as a control. Figure presented full-length gels.

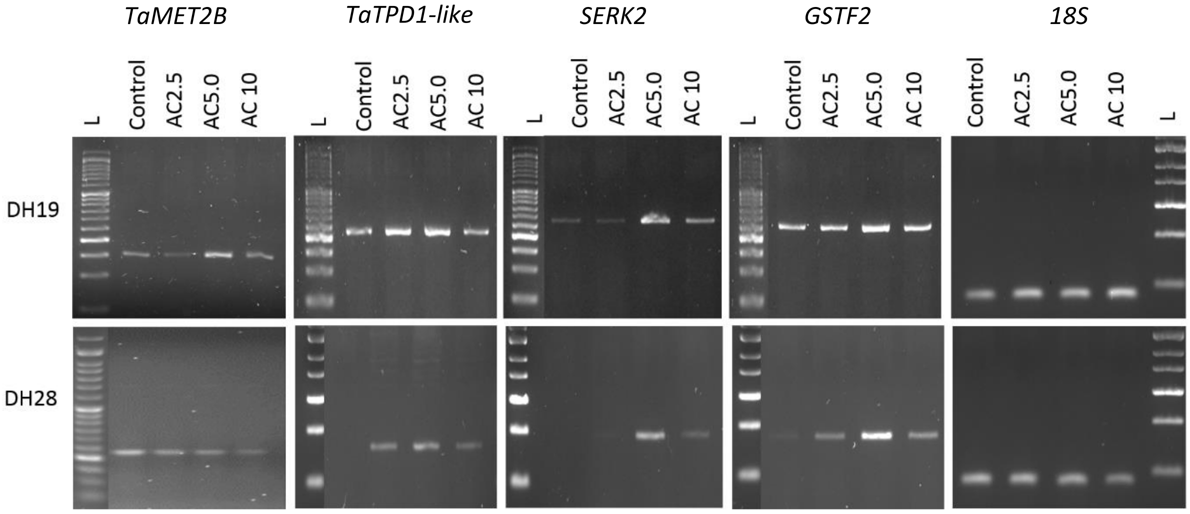

Supplementary Table S5. Primers used in this study.

Primer name	Sequence 5' to 3'	References
TaMET2B_F	TGTGCCACCCCCTCTTGC	Thomas et al.[65]
TaMET2B_R	CAGTTCATGCCAGCCATGC	Thomas et al.[65]
TaTPD1-like_F	AAGTTTCAGCGTCTTCCTCGCT	Żur et al.[12]
TaTPD1-like_R	TCGATGCAGGTGTTGGTGAACT	Żur et al.[12]
SERK2_F	AGCTTCGATTCCTCCGTCTT	Żur et al.[12]
SERK2_R	AGGCACCTGCTGATTGAGTT	Żur et al.[12]
GSTF2_F	CGGCAAAGCTGACGAATCTGTT	Żur et al.[12]
GSTF2_R	ACTTCTCTGCCTTCTTTCCGAACC	Żur et al.[12]
18SrDNA_F	CGGCTACCACATCCAAGGAA	Żur et al.[12]
18SrDNA_R	TGTCACTACCTCCCCGTGTCA	Żur et al.[12]
